# Establishment of an erythroid progenitor cell line capable of enucleation achieved with an inducible c-Myc vector

**DOI:** 10.1186/s12896-019-0515-9

**Published:** 2019-04-15

**Authors:** Steven Mayers, Pablo Diego Moço, Talha Maqbool, Pamuditha N. Silva, Dawn M. Kilkenny, Julie Audet

**Affiliations:** 1 Department of Chemical Engineering and Applied Chemistry, University of Toronto, Toronto, Canada; 2 Institute of Biomaterials and Biomedical Engineering (IBBME), University of Toronto, Toronto, Canada

**Keywords:** Erythroid progenitor cell, Induced proliferation, Red blood cell, c-Myc, Enucleation, Cell manufacturing

## Abstract

**Background:**

A robust scalable method for producing enucleated red blood cells (RBCs) is not only a process to produce packed RBC units for transfusion but a potential platform to produce modified RBCs with applications in advanced cellular therapy. Current strategies for producing RBCs have shortcomings in the limited self-renewal capacity of progenitor cells, or difficulties in effectively enucleating erythroid cell lines. We explored a new method to produce RBCs by inducibly expressing c-Myc in primary erythroid progenitor cells and evaluated the proliferative and maturation potential of these modified cells.

**Results:**

Primary erythroid progenitor cells were genetically modified with an inducible gene transfer vector expressing a single transcription factor, c-Myc, and all the gene elements required to achieve dox-inducible expression. Genetically modified cells had enhanced proliferative potential compared to control cells, resulting in exponential growth for at least 6 weeks. Inducibly proliferating erythroid (IPE) cells were isolated with surface receptors similar to colony forming unit-erythroid (CFU-Es), and after removal of ectopic c-Myc expression cells hemoglobinized, decreased in cell size to that of native RBCs, and enucleated achieving cultures with 17% enucleated cells. Experiments with IPE cells at various levels of ectopic c-Myc expression provided insight into differentiation dynamics of the modified cells, and an optimized two-stage differentiation strategy was shown to promote greater expansion and maturation.

**Conclusions:**

Genetic engineering of adult erythroid progenitor cells with an inducible c-Myc vector established an erythroid progenitor cell line that could produce RBCs, demonstrating the potential of this approach to produce large quantities of RBCs and modified RBC products.

**Electronic supplementary material:**

The online version of this article (10.1186/s12896-019-0515-9) contains supplementary material, which is available to authorized users.

## Background

Red blood cells (RBCs) are an ideal platform for novel cellular therapies as they are physically stable, universally biocompatible (type O, Rhesus factor negative), contain no nucleus, and can potentially be packed and coated with biologically active molecules. It has already been shown that RBCs can be engineered to carry therapeutic molecules in multiple ways [1–3], and pre-clinical studies have shown proof of concept for RBC based immunotherapy [3–5]. Production of human RBCs from hematopoietic stem cells has been achieved with 60,000 fold expansion from cord blood stem cells including safe transfusion into human patients, signifying the potential for the clinical translation of in vitro cultured RBCs [6].

RBCs are produced by erythropoiesis, which is an erythropoietin (EPO)-dependant cell development process in which a nucleus-containing erythroid precursor is differentiated to become a hemoglobin-containing enucleated RBC [7]. The earliest committed erythroid progenitor is the burst forming unit-erythroid (BFU-E) [8]. BFU-Es differentiate to form CFU-Es (c-kit^+^CD71^high^Ter119^−^) [9–11], which usually divide three to five times over two to 3 days as they hemoglobinize, and undergo a decrease in cell size [8]. As these cells develop, they exit the cell cycle, repress transcription, condense their chromatin, and finally extrude their nucleus to form a reticulocyte [12]. After about 2 days, the reticulocyte shows loss of reticulin as it terminally differentiates into a RBC [13].

Induced proliferation of erythroid progenitor cells is a promising strategy for producing RBCs that has been explored with some success. It was shown that the introduction of HPV16-E6/E7 genes into erythroid progenitor cells expressed by an inducible promoter could achieve enhanced proliferation while retaining the ability for cells to terminally differentiate [14, 15]. This approach was applied to adult bone marrow-derived cells and although a robust RBC production platform was reported, significant cell losses were observed during differentiation (approx. 80%), and of those viable cells, enucleation was observed in about 25% of the remaining cells [14]. Another group has reported the creation of inducibly proliferating erythroid (IPE) progenitor cells from human embryonic stem (ES) cells by over-expression of both c-Myc and bcl-xl [16]. In this approach, with c-Myc removal, cells hemoglobinized, but with only 0.36% enucleation efficiency. Bcl-xl expression suppresses p53, which is an regulator of apoptosis and genomic integrity [17], potentially limiting the genomic stability and developmental potential of these cells.

Induced proliferation of many cell types has been shown by ectopic expression of the transcription factor Myc alone [18–20]. Expression level is important, where supraphysiological levels have been shown to be required for driving induced erythroid progenitor cell proliferation [18]. The transient up-regulation of v-Myc has been shown to induce reversible proliferation of primary neural progenitors with no observed tumorigenic impact [19]. In a β-cell in vivo model, the selective expression of c-Myc induced a proliferative state accompanied by broad reversible gene expression changes, indicating the reversible nature of c-Myc induced proliferation [20].

The transcription factor c-Myc activates many genes involved in cell proliferation, promoting growth and inhibiting cell cycle arrest [21–24]. C-Myc is a G_0_/G_1_ transition regulator which promotes cell cycle entry [25]. Over-expression of c-Myc can induce apoptosis through a p53 tumour suppression pathway, which eliminates cells that are inappropriately bypassing the G_1_-S checkpoint [26]. The effect of ectopic c-Myc expression on apoptosis depends on its level of expression [24], as well as the state of the cell and its physiological status [27]. In vitro*,* the effect of c-Myc on bcl-2 family proteins and cytochrome C release may be blocked by the survival factor insulin like growth factor 1 (IGF-1) [28]. Also, apoptosis induced by c-Myc over-expression can also be avoided by complementary signal transduction pathways that result from the presence of mitogens [29]. C-Myc-induced sensitization to apoptosis presents a challenge when inducing proliferation, where the ideal expression would be just enough to induce proliferation accompanied by sufficient mitogenic survival signals to prevent triggering apoptosis.

C-Myc has been shown to positively regulate histone acetyl transferases (HATs) which expose DNA through chromatin remodelling [30]. In erythroid cell development, histone deacetylation, which reverses HAT activity, is critical for chromatin condensation and enucleation [18]. In erythroid cells in which c-Myc has been ectopically expressed, HAT up-regulation results in an inhibition of nuclear condensation [18]. These observations outline the importance of complete removal of c-Myc expression to allow for histone deacetylation, chromatin condensation, and enucleation of erythroid progenitors.

In attempts to develop a new method to produce large quantities of RBCs, inducible over-expression of c-Myc in primary erythroid progenitors was investigated. The proliferative capacity of modified cells expressing ectopic c-Myc was evaluated, as well as their ability to terminally differentiate upon ectopic expression removal. Our goal was to establish an erythroid progenitor cell line capable of extensive self renewal and terminal differentiation into enucleated RBCs.

## Results

### Tightly controlled ectopic expression of functional c-Myc

**Fig. 1:**
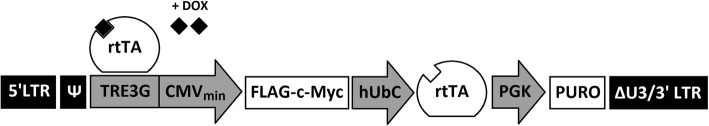
Simplified DNA plasmid map of lentivirus transfer vector TRE3G-cMyc. The vector is a third-generation lentivirus transfer vector containing the third-generation tetracycline responsive element (TRE3G) controlling the expression of FLAG tagged c-Myc (mouse) transcription factor, constitutive expression of the rtTA, and a puromycin (puro) resistance gene. Depicted is the rtTA transcription factor activated by soluble dox binding to the TRE3G promoter

An all-in-one lentiviral gene transfer vector (Fig. 1 and Additional file 1: Figure S1) was developed to achieve dox-inducible expression of the transcription factor c-Myc in primary cells. The vector contained the c-Myc (mouse) gene with an N-terminus FLAG-tag under the third-generation doxycycline/tetracycline responsive element (TRE3G) transcriptional promoter which has minimal background expression [31]. To achieve full gene expression control with a single vector, the reverse tetracycline transactivator (rtTA) gene was included in the vector under constitutive expression. The gene vector also contains a puromycin resistance gene under constitutive expression to allow for selection of genetically modified cells by culturing them in media containing puromycin.

**Fig. 2:**
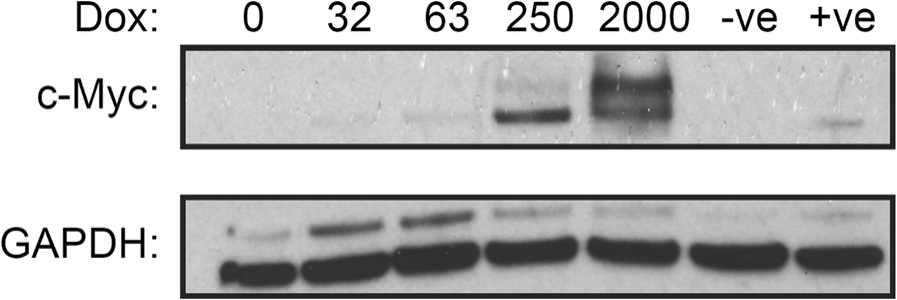
Western blot of c-Myc knock-out cells (HO15.19) stably expressing the TRE3G-cMyc transfer vector. Cells were plated at 2.0 × 10^5^ cells/ml in various dox concentrations with 2.0 ml per well in a 6-well plate. Negative (−ve) control (non-modified HO15.19), and positive (+ve) control (wild type TGR-1) cells were included. Cells were cultured for 24 h to allow for dox induced c-Myc expression, then cellular proteins were analysed by western blot using anti-c-Myc and anti-GAPDH antibodies

**Fig. 3:**
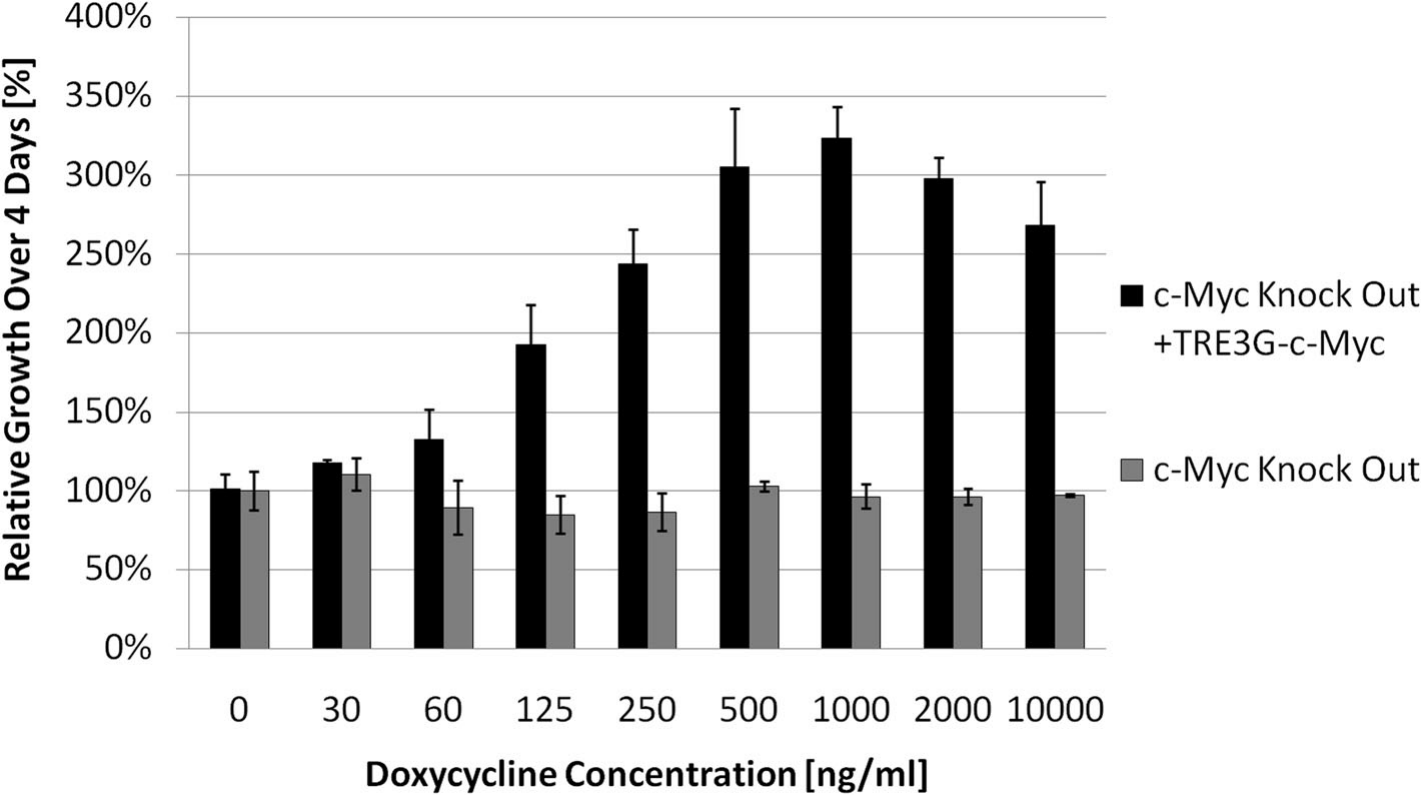
Functional validation of TRE3G-cMyc transfer vector in c-Myc knock-out cells HO15.19. Cells modified and purified with puromycin were cultured for 4 days with various dox concentrations. Growth for all conditions was measured relative to the no vector control with no dox. Relative growth was measured as relative fluorescence using the viability assay reagent AlamarBlue at 4 days which assumes AlamarBlue conversion is linearly proportional to cell number

This all-in-one gene transfer vector was validated in the c-Myc^−/−^ knock-out fibroblast cell line HO15.19 which grows slower without c-Myc [32]. A purified population of HO15.19 cells, carrying the TRE3G-cMyc transfer vector, had a dox-dependant expression of c-Myc. This was shown by western blot (Fig. 2), and a cell proliferation assay (Fig. 3). Together, these assays showed a clear dynamic range of dox-induced c-Myc expression that increased the growth rate of vector containing cells proportionally to dox concentration. Negligible c-Myc background expression with no dox was confirmed by western blot, and the growth rate of modified cells with no dox matching the knock out cell line (Additional file 1: Figure S2). The western blot confirmed that the size of the c-Myc protein matched the endogenously produced transcription factor (50 kDa). At high dox concentrations a larger protein band is visible which is consistent with a phosphorylated form of c-Myc [33].

### Induced proliferation of c-Myc transduced hematopoietic progenitor cells

**Fig. 4:**
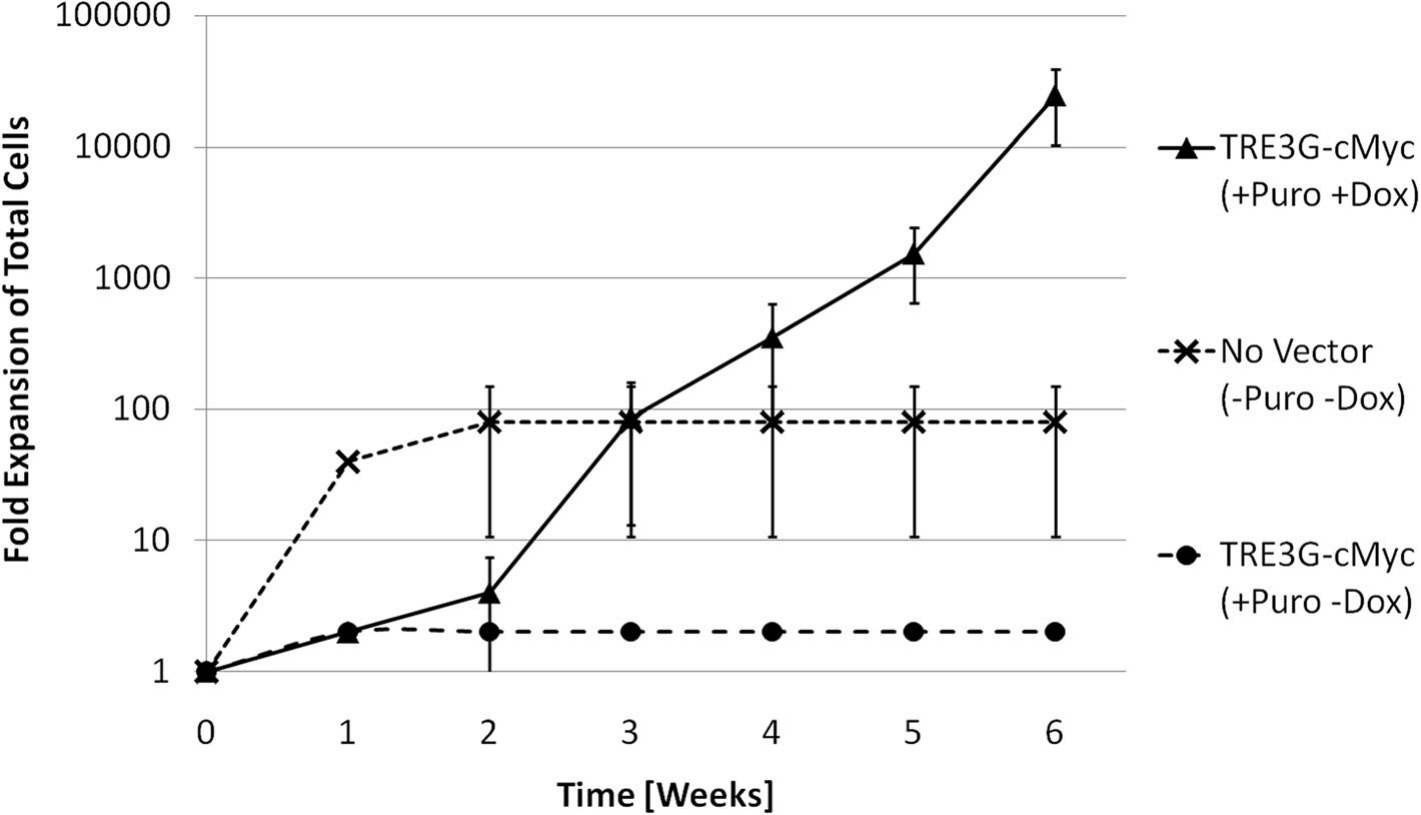
Induced proliferation of lin^−^Ter119^−^Mac-1^−^Gr-1^−^c-Kit^+^CD71^(low/−)^ mouse bone marrow cells by over-expression of the transcription factor c-Myc. Shown are average measurements from 3 separate in vitro experiments where cells were modified by stable gene transfer of the TRE3G-cMyc vector, cultured in puromycin to eliminate non-modified cells (except wild type condition), then cultured with 2000 ng/ml dox for expression of the vector gene of interest or no dox as a control. Passage number and split ratio were used to determine fold expansion of total cells from the starting well and shown for 6 weeks of culture

Three separate independent experiments were done on different preparations of primary mouse bone marrow cells purified to have the surface receptor profile lineage negative (Lin^−^)Ter119^−^Mac-1^−^Gr-1^−^c-Kit^+^CD71^(low/−)^ (Additional file 1: Figure S3), which is known to be enriched with BFU-Es [9]. The presence of BFU-Es was confirmed by colony forming cell (CFC) assay (Additional file 1: Figure S4). Cells were either genetically modified with the TRE3G-cMyc vector, or treated with a phosphate buffered saline (PBS) control, then cultured in the presence of growth factors, with or without dox, and analysed over a period of 6 weeks (Fig. 4). For the PBS control condition, the total number of cells increased rapidly to 100-fold over a period of two weeks and then stopped proliferating. For both conditions where cells were modified with the TRE3G-cMyc vector, substantial cell death occurred as a result of the puromycin selection step. Modified cells with no dox present showed only a modest (2-fold) net increase, which was still substantial growth considering the losses from puromycin selection. In contrast, in cultures with dox, and therefore ectopic c-Myc expression, cell number increased extensively after the puromycin selection step; cells proliferated continuously for at least 6 weeks reaching a 22,000-fold net expansion.

**Fig. 5:**
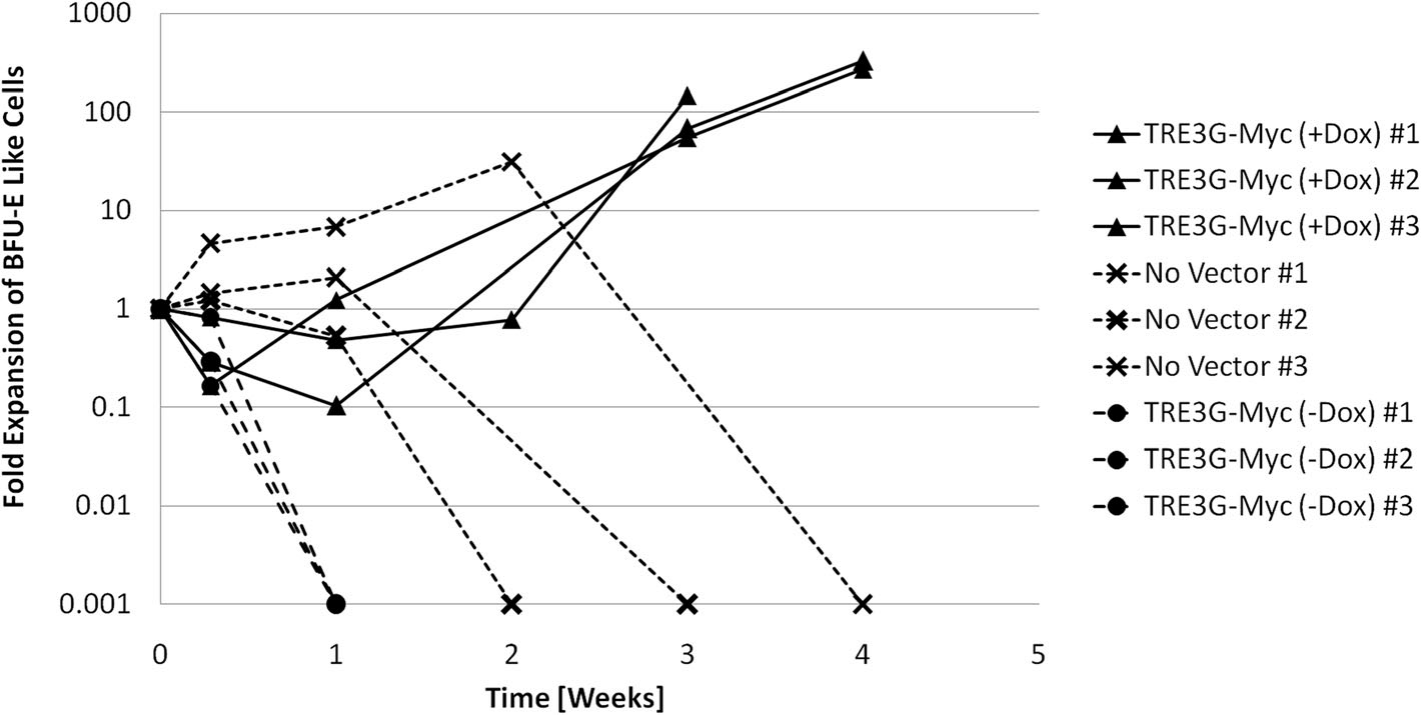
Induced proliferation of ‘BFU-E like’ cells by over-expression of the transcription factor c-Myc. Shown are measurements from 3 separate in vitro experiments where cells were modified by stable gene transfer of the TRE3G-cMyc vector, cultured in puromycin to eliminate non-modified cells (except wild type condition), then cultured with 2000 ng/ml dox for expression of the vector gene of interest or no dox as a control

To further investigate c-Myc-induced proliferation, fractions of the cell cultures were analysed using CFC assays to assess for clonogenic erythroid progenitor cells. Colonies derived from modified cells expressing c-Myc ectopically were atypical for mouse bone marrow hematopoietic cells, containing what appeared to be a large number of undifferentiated (blast) cells even after two weeks of incubation in methylcellulose (with dox and growth factors). The appearance of these colonies resembled human BFU-Es [34] (Additional file 1: Figure S5), and were described as ‘BFU-E like’ colonies. As shown in Fig. 5, BFU-E-enriched cells transduced with the TRE3G-cMyc vector cultured in the presence of dox showed an increase in ‘BFU-E like’ colonies that reached at least a 100-fold net expansion over a period of 3 weeks. This sustained expansion was not observed in modified cultures without dox, or non-transduced cells clearly showing the positive effect of ectopic c-Myc expression on self-renewal of CFC. Greater proliferative potential and CFC expansion was achieved in all three experiments where c-Myc was ectopically expressed compared with all control conditions.

### Isolation of IPE cells

After 3.5 weeks of culture, a purified population of IPE cells was isolated by flow cytometry with the surface marker expression profile Mac-1^−^Gr-1^−^c-Kit^(high/+)^CD71^+^ (Additional file 1: Figure S6) which in enriched in CFU-Es [9, 35]. For the other cell isolation experiments, the cultures contained a majority of Mac-1^+^ and Gr-1^+^ positive cells after 3.5 weeks (Additional file 1: Figure S7) and erythroid cells were too rare to successfully isolate and culture. The isolated ‘CFU-E like’ cell line was cultured further in the presence of growth factors (IPE Media) and 2 μg/ml dox for at least an additional 23 days after isolation. The cells maintained expression of CD71 and c-Kit without giving rise to Gr-1^+^ or Mac-1^+^ cells (Additional file 1: Figure S6), indicating that c-Myc was capable of inducing self-renewal of erythroid progenitors far beyond the expected self-renewal capacity of only a few divisions [36].

### Cytokine dependence of IPE cells typical of erythroid cells

**Fig. 6:**
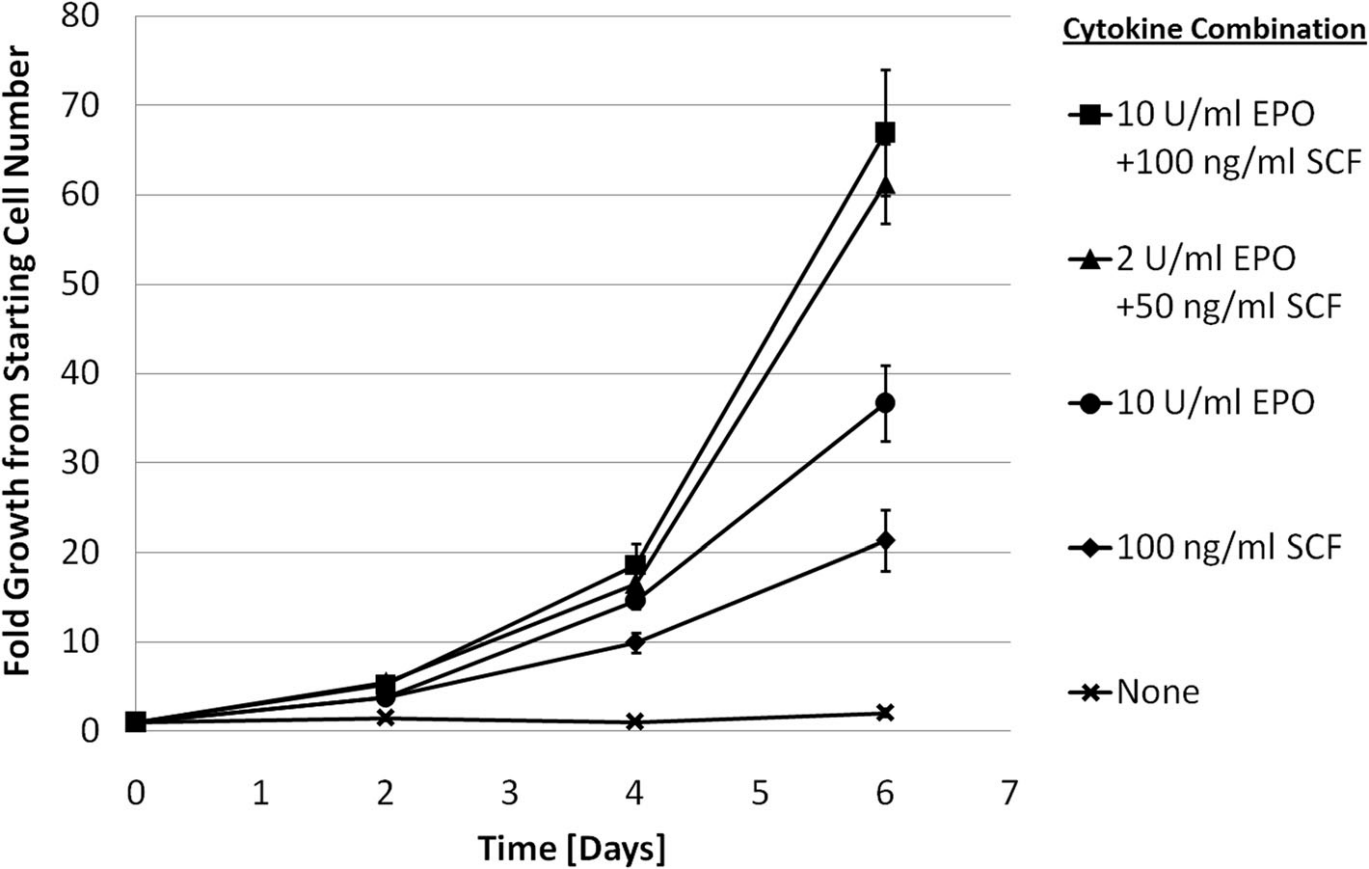
Cytokine dependence of IPE cell expansion. IPE cells were plated at 2.5 **×** 10^5^ cells/ml in 200 μl IPE base media (without EPO and SCF) and cultured for 6 days with various combinations of EPO and SCF cytokines. Cells were passaged 1:4 every 2 days, and each data point is the average of three technical replicates

IPE cells responded to both erythropoeitin (EPO) and stem cell factor (SCF), each cytokine having a positive effect on cell proliferation (Fig. 6). Culture with both EPO and SCF achieved the greatest proliferation of cells, reaching over a 60-fold expansion after 6 days of culture. In contrast, significant cell death was observed for cells cultured in the absence of both EPO and SCF.

### Colony-forming ability of IPE cells

**Fig. 7:**
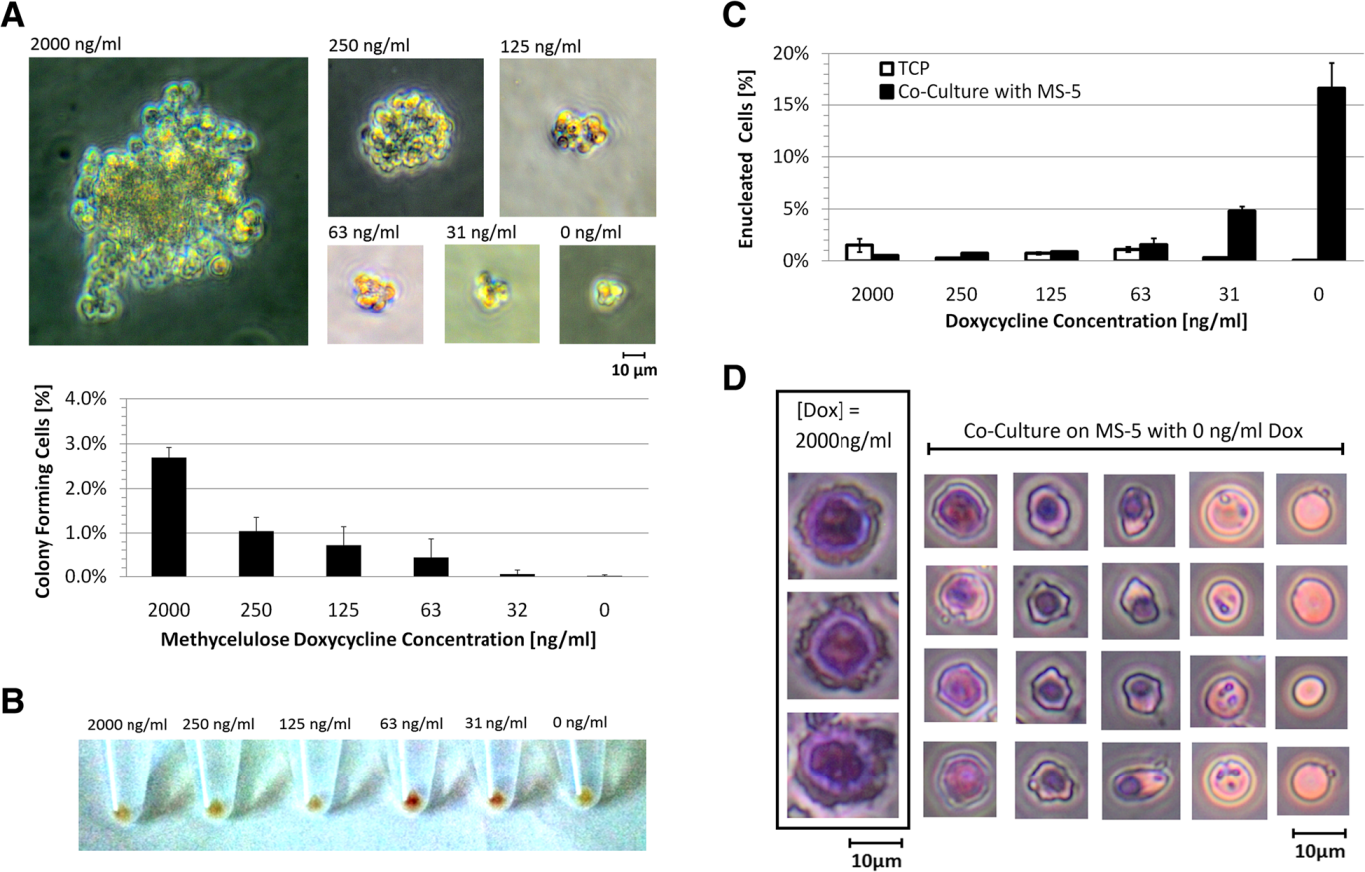
IPE cell colony forming characterization and developmental potential. **a.** (TOP) Colony forming assays of IPE cells at various concentrations of dox. Images of representative colonies formed after 1 week of culture in various dox concentrations**.** (BOTTOM) The percentage of cells that formed colonies for each concentration of dox after 7 days. **b.** IPE cell pellets imaged after 2 days of culture on TCP in IPE media with various dox concentrations. **c.** Average enucleated non-granular cell percentage after 4 days for triplicate differentiation cultures either on TCP or on a confluent layer of mouse stromal cells (MS-5). **d.** IPE cells and differentiated IPE cells stained using the Giemsa histology stain to allow for the identification of the cytoplasm and nuclear material. IPE cells were differentiated by co-culture on MS-5 for 4 and 8 days with 50% media exchange every 2 days. All images were taken at 400× magnification and scaled equivalently to show size variation between developmental stages

CFC assays were performed on IPE cells in the presence of varying concentrations of dox. Both the frequency and the cell output (colony size) per clonogenic cell was dependent on the concentration of dox (Fig. 7a), where higher dox concentrations resulted in a higher frequency of colony formation and larger colonies. The maximum CFC frequency obtained was low (2.5% at 2000 ng/ml dox) which suggests that the cell population was heterogeneous. This could be because erythroid progenitors at different stages of differentiation are self-renewing in culture, or a small population of BFU-E/CFU-E progenitors are self-renewing to a certain extent but also differentiating to some degree. The colonies formed at high concentrations of dox appeared largely composed of clustered undifferentiated (blast) cells. The phenotype of the colonies formed with dox concentrations of 63 ng/ml and 125 ng/ml appeared very similar to CFU-Es, comprising small clusters of small cells. Interestingly, at 0 ng/ml or 32 ng/ml dox, the few colonies that formed appeared unhealthy and underdeveloped with too few cells to be considered a CFU-E [37].

### Hemoglobinization with reduced c-Myc expression

Culture of IPE with reduced ectopic c-Myc expression resulted in red pigmentation observed in cell pellets after centrifugation, indicating the presence of hemoglobin [15]. This development of red pigmentation (Fig. 7b) was more pronounced for cells cultured at an intermediate level of dox (63 ng/ml dox) and c-Myc expression, suggesting that a reduction (and not complete removal of ectopic c-Myc expression) was optimal for promoting differentiation into hemoglobin-producing cells. The presence of hemoglobin was confirmed by tetramethylbenzidine staining [38–40] (Additional file 1: Supplementary Methods and Figure S8). These results indicate that IPE cells can produce hemoglobin to some degree.

### Enucleation potential

To promote IPE cell differentiation and enucleation, dox was removed from the culture media to stop ectopic c-Myc expression in attempts to restore normal erythroid development. The enucleation methods included co-culture on a confluent layer of mouse stromal cells (MS-5) which has been shown to promote enucleation of erythroid cells in vitro [41]. After 4 days of co-culture on MS-5 cells at 0 ng/ml dox, a clear population of nucleus-free cells was observed which was quantified to be 17 ± 2% of the total cells when measured by flow cytometry (Fig. 7c). The same high levels of enucleated cells was not observed in cultures without MS-5, where after 4 days of culture there was no significant increase in proportion of enucleated cells.

The enucleation potential of IPE cells was further assessed using the Giemsa histology stain combined with cytospin (Additional file 1: Supplementary Methods) in which erythroid cells at different stages of normal erythropoeisis are distinguishable (Fig. 7d) [42]. After dox removal, nuclear condensation and polarisation was observed, indicated by the presence and position of the cell nucleus [12]. Enucleated reticulocytes were visible based on their lack of nucleus and presence of residual RNA [43]. Enucleated cells without visible residual RNA were also present which correspond to terminally differentiated RBCs.

### Decrease in cell size

The size of enucleated cells produced from IPE cells before and after culture on MS-5 with 0 ng/ml dox were measured with phase contrast microscopy and compared with fresh mouse RBCs. The diameter of IPE cells decreased from 14.5 ± 0.4 μm to 6.7 ± 0.9 μm after enucleation, which is similar to wild type RBCs which have a diameter of 5.4 ± 0.5 μm (Additional file 1: Figure S9).

### IPE cells with intermediate levels of ectopic c-Myc expression

**Fig. 8:**
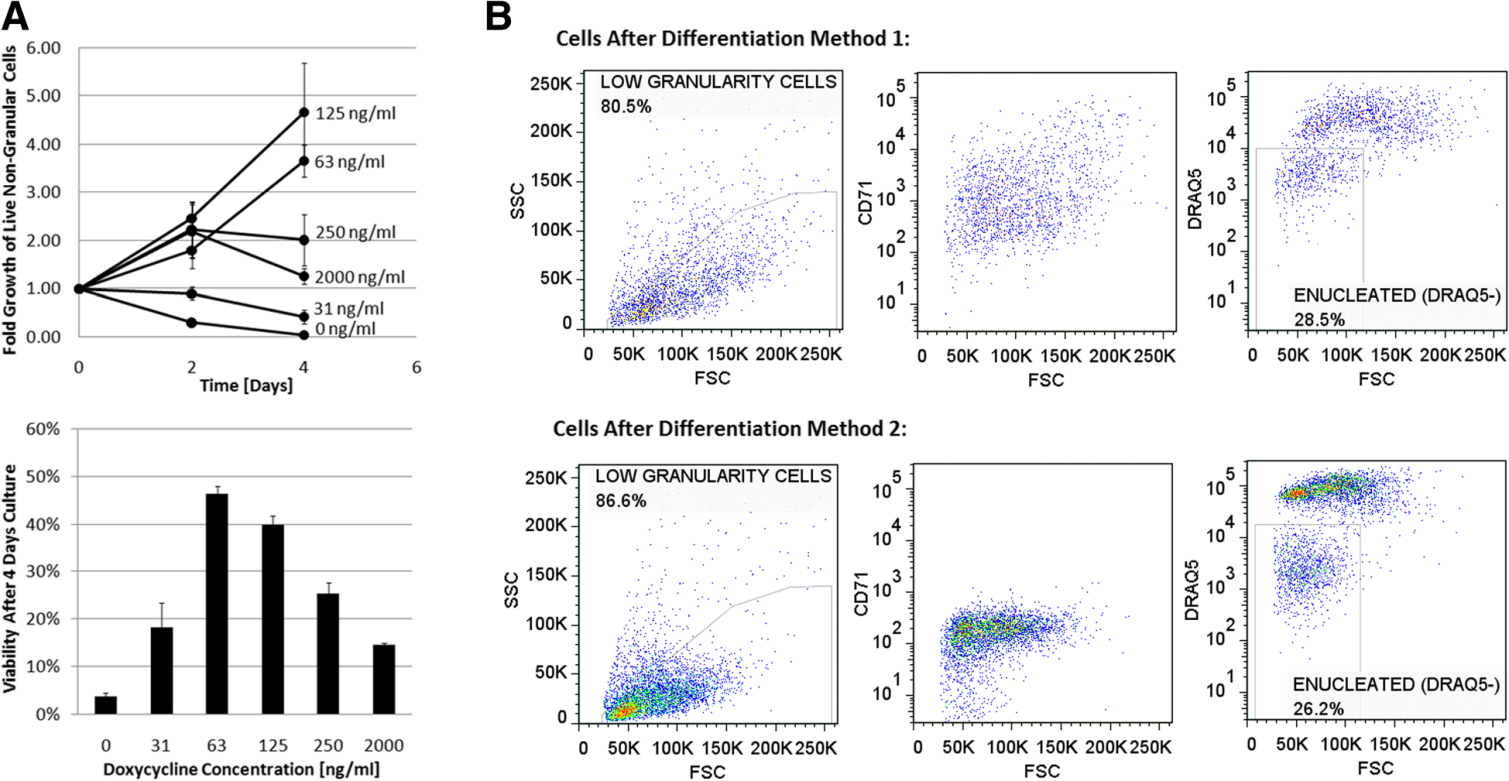
IPE cell dox dependence assay and optimized differentiation results **a**. (TOP) Fold growth, and (BOTTOM) cell viability of IPE cells cultured with various dox concentrations on TCP. For each time point triplicate 200 μl cultures of IPE cells were plated at 5 × 10^5^ cells/ml in a single well of a 96-well plate. Cells were cultured under static conditions at 37 °C and 5% CO_2_ without passage, media change, or agitation. For each measurement, cells were stained with 7AAD and mixed with cell counting beads for analysis with flow cytometry. **b**: IPE cells after two different differentiation methods. Method 1 (TOP): 4 days of co-culture on MS-5 with 0 ng/ml dox. Method 2 (BOTTOM): 2 days of culture on TCP with 125 ng/ml dox, and then 4 days of co-culture on MS-5 with 0 ng/ml dox. For each condition the final cells were analyzed using flow cytometry measuring forward (FSC) and side (SSC) scatter, 7AAD, CD71 (transferrin receptor), and DRAQ5 (nuclear stain)

By culturing IPE cells on tissue culture plastic (TCP) at various concentrations of dox, it was found that cells transition to a unique cell phenotype when exposed to intermediate concentrations of dox (between 63 ng/ml and 125 ng/ml) as shown in Fig. 8a Reducing the dox concentration from 2000 ng/ml to 125 ng/ml promotes transition to a different cellular phenotype compared with both high and no dox. This cell phenotype is characterized by a lower proliferative rate, and better cell survival than when dox is completely removed (Fig. 8a). In this assay, cells were plated for 4 days at a high seeding density and, as a result, cultures with high levels of dox (250 ng/ml and 2000 ng/ml) reached excessive cell densities (5.0 × 10^6^ cells/ml) and significant cell death was observed which was atypical of routine passage.

### IPE cell development by gradual reduction of ectopic c-Myc expression

A two-stage differentiation method was investigated to understand if a transitional stage with intermediate levels of ectopic c-Myc expression, before complete dox removal, could improve the development of IPE cells into enucleated RBCs. Cells cultured for 2 days at a reduced level of ectopic c-Myc expression (125 ng/ml dox) and subsequently with complete removal (0 ng/ml dox) in co-culture with MS-5 cells for 4 days appeared to undergo maturation more readily than those with no intermediate stage of ectopic expression (Fig. 8b). The transferrin receptor CD71 was completely lost in the two-stage approach in contrast to only moderate loss in the immediate transition approach. A major difference was the presence of a large homogeneous population of very small, non-granular nucleus containing (DRAQ5-positive) cells with the two-stage approach. As well, this two-stage process resulted in a more homogenous population of small, low granularity, and nucleus- free cells which seems to more closely recapitulate normal erythropoiesis.

## Discussion

### Increased progenitor cell proliferation with c-Myc

The ectopic expression of high levels of c-Myc consistently induced extensive proliferation of primary hematopoietic progenitor cells, far exceeding the proliferative capacity of wild type cells. Ectopic expression of c-Myc promoted extensive cell proliferation (Fig. 4), and self-renewal of CFC (Fig. 5) with a unique morphology which is reminiscent of human BFU-E colonies (Additional file 1: Figure S5). The isolation of IPE cells modified with c-Myc alone is consistent with previous reports of c-Myc inducing proliferation of primary erythroid mouse cells [18], but differs from published work in human erythroid progenitor cells, where c-Myc-induced proliferation was observed to stop after a few weeks [16]. This discrepancy may be explained by the difference in species, but may also be the result of differences in the level of ectopic c-Myc expression achieved, the cell culture strategy, the medium composition, or a combination of these factors. In this work, intercellular signaling was optimized by maintaining the cellular aggregates that formed during routine cell passage. Also, in this work media was supplemented with IGF-1, which is known to suppress p53 induced apoptosis [28, 44], and this may have promoted cell survival. The genomic stability of IPE cells was not determined as karyotype analysis was not performed. However, mutagenesis is not considered to be the mechanism of c-Myc-induced proliferation. The reproducibility of increasing proliferative potential with c-Myc expression suggests that increased proliferative capacity is the result of increased transcription factor-associated gene expression and not a reproducible c-Myc-induced genomic mutation (mutagenesis is expected to be more random and less reproducible). The susceptibility of IPE cells to mutagenesis in long term culture should however be assessed if the use of IPE cells requires a stable cell line.

### Isolating IPE cells

Although ectopic c-Myc expression enhanced cell expansion in all three experiments, isolation of induced proliferating erythroid cells was only achieved in one experiment. In the experiments where erythroid cell lines could not be isolated, the majority of the cells had become Gr-1 or Mac-1 positive, indicating the presence of predominantly macrophage and granulocyte progenitors. Although the starting population was enriched for BFU-Es, mixed hematopoietic colonies were observed, and the culture conditions included SCF which supports the survival and proliferation of progenitor cells in other lineages. Challenges in isolating erythroid cell lines may also be the result of inhibitory factors in the culture media. IPE media contained FBS which may have contained transforming growth factor beta (TGF-β) [45], a known inhibitor of erythropoiesis [46]. The potential contribution of inhibitory factors is supported by the observation that the expansion of wild type BFU-Es was lower than expected [9]. Regardless of the challenges encountered when culturing purified erythroid cells, IPE cells were successfully generated, isolated, and further characterized.

### IPE cells respond to cytokines

The individual and combined effects of EPO and SCF cytokines on IPE cell proliferation shown in Fig. 6 are similar to what has been reported for non-transduced mouse CFU-E [35, 47] but with much higher yields. In the absence of both EPO and SCF, there was significant cell death which is also observed in wild type cell cultures [35]. The observed cytokine-mediated cell survival is an indication of conserved apoptosis regulation in IPE cells. Therefore, it seems likely that evasion of c-Myc-induced apoptosis during IPE proliferation is the result of complementary signal transduction pathways affecting cell survival [29], and not a mutation affecting the cells ability to undergo apoptosis. This is supported by the known role of both SCF and EPO in activating bcl-xl [48–50] which binds to bim and blocks its apoptotic activity [51]. Inducing proliferation with over-expression of c-Myc without the apoptosis regulation disrupting genetic modifications used in other approaches [16, 52] is likely to result in a more stable cell line, with its genome still monitored by the p53 [17].

### IPE cell differentiation into RBCs

IPE cells were reproducibly shown to differentiate with a reduction of ectopic c-Myc expression, signifying the reversibility of c-Myc-induced proliferation. As shown in Fig. 7, upon reducing ectopic c-Myc expression, IPE cells hemoglobinized, decreased in cell size and enucleated. Enucleation was best achieved with co-culture with MS-5, which has been shown to promote erythroid enucleation [41]. Measured enucleation of mouse erythroid cells modified with c-Myc alone allowed for 17% of cells to become enucleated, which far exceeds reported enucleation results reported from human cells modified with both c-Myc and bcl-xl where the enucleated fraction of differentiated cells was reported to be just 0.36% [16]. An improved differentiation strategy based on staged c-Myc expression was developed where cells hemoglobinized and formed colonies in methylcellulose in an optimal level when subjected to an intermediate level of ectopic c-Myc expression. It was found that some level of ectopic c-Myc expression was required for survival which is in agreement with previous reports suggesting that cells with c-Myc over-expression can become ‘addicted’ to c-Myc and where turning off conditional c-Myc can cause apoptosis [53]. By staging the removal of ectopic c-Myc expression, better development through erythropoiesis was observed (Fig. 8b). The enucleated RBC fraction contained predominantly small non-granular cells, and was more homogenous than RBCs produced by a one-step removal. The remaining nucleus-containing cells were also more homogenous, with a unique cluster of small, non-granular, CD71^−^ viable cells, indicating the potential for further enucleation in cultures with longer incubation times. Further work is required to optimize the staged differentiation approach, but these preliminary results indicate it promoted erythroid maturation, establishing a new strategy to produce RBCs from induced proliferating erythroid progenitor cells.

## Conclusions

A functionally validated all-in-one lentiviral gene transfer vector was developed and made it possible to achieve a tightly controlled dox-inducible expression of a ‘FLAG’ tagged c-Myc transcription factor. Genetic engineering of primary bone marrow-derived erythroid progenitor cells with this vector enabled the creation of IPE cells. These cells proliferated beyond the limited potential of wild type cells and their number increased over a period of at least 6 weeks. These cells maintained a surface receptor profile and cytokine dependence similar to wild type CFU-Es.

IPE cells were shown to maintain their developmental potential with ectopic c-Myc removal; they produced hemoglobin, decreased in size and enucleated. It was discovered that transition of IPE cells to low levels of ectopic expression, instead of complete removal, allowed for CFU-E like colony formation in methylcellulose, greater hemoglobinization and greater cell survival. Considering that complete removal of c-Myc was important for chromatin condensation and enucleation, an optimized two-stage differentiation strategy was developed, and was shown to promote IPE cell differentiation. Using this two-stage approach, after 6 days of culture, a final cell population was obtained which contained 16% ± 3% of enucleated cells. Further work is required to optimize this differentiation protocol to achieve higher enucleation efficiency.

This work demonstrates a new method to create inducibly proliferating erythroid progenitor cells which can maintain their ability to subsequently produce enucleated RBCs. This method is potentially useful for the production of large quantities of clinically-compatible enucleated cells. Furthermore, this strategy for RBC production presents a valuable platform to produce engineered enucleated cells expressing and containing biological molecules for novel cellular therapies.

## Methods

### TRE3G-c-Myc transfer vector molecular cloning and lentivirus production

The reverse tetracyclin responsive element (rtTA) with the human Ubiquitin C (hUbC) constitutive promoter from the FUW-M2rtTA plasmid (a gift from Rudolf Jaenisch, Addgene ref.: 20342) [54] was amplified with primer set 1 (Additional file 1: Supplementary Methods) to create the DNA fragment NotI-HUbC-rtTA-EcoRV. This was cloned into the pENTR1 Gateway Entry plasmid (a gift from Eric Campeau, Addgene ref.: 19364) [55] resulting in the pENTR1-GFP-HUbC-rtTA plasmid. Template DNA for mouse c-Myc (Isoform 2) was amplified from TetO-FUW-cMyc (a gift from Rudolf Jaenisch, Addgene ref.: 20324) [56] using primer set 2 (Additional file 1: Supplementary Methods) introducing a FLAG epitope (Peptide: DYKDDDDK) on the N–Terminus of c-Myc gene, creating the DNA fragment SacI-FLAG-c-Myc-NotI. This was cloned into the pENTR1-GFP-HUbC-rtTA creating the pENTR1-FLAG-cMyc-HUbC-rtTA plasmid. This was then cloned into the pLenti CMVTRE3G Puro Destination plasmid (a gift from Eric Campeau, Addgene ref.: 27565) [55] creating the final transfer vector pLenti-TRE3G-FLAG-cMyc-HUbC-rtTA-PGK-Puro plasmid (TRE3G-cMyc). Transfer vectors were packaged in HEK293T cells (ATCC, ref.: CRL-3216) with plasmids psPAX2 and VSV-G (gifts from Didier Trono, Addgene refs: 12260 and 12259), then concentrated by ultracentrifugation, and stored at − 80 °C. Titres were quantified using puromycin in colony-forming assays [57]. Additional information about lentivirus production and concentration is available in Additional file 1: Supplementary Methods.

### Vector validation assay with c-Myc^−/−^ cell line

Rat fibroblast cells TGR-1 [32], the c-Myc^−/−^ knock out variant HO15.19 [32], and HO15.19 modified with the TRE3G-cMyc vector were passaged under normal cell culture conditions (Additional file 1: Supplementary methods) and plated at low densities of 1000 cells per well in a 96-well plate (Sarstedt, ref.: 83.1835) at various dox concentrations. After 4 days, to detect relative cell density cells were incubated in 5% *v*/v AlamarBlue (AbDSerotec, ref.: BUF012B) for 1 h under normal culture conditions. The quantity of reaction product was detected using a SpectraMAX GeminiXS (Molecular Devices) at an excitation frequency of 530 nm and detection above 590 nm [58]. Relative growth was a direct comparison of reaction products and doubling times were calculated using an exponential growth model (Additional file 1: Supplementary Methods).

### Western blot

Cells were collected in 1X phosphate buffered saline (PBS), centrifuged and re-suspended in lysis buffer (1% Triton-X-100, 100 mM sodium chloride, 50 mM HEPES, 5% glycerol, protease inhibitor mixture and PhosphoSTOP phosphatase inhibitor (Roche Applied Science) as previously described [59]. Concentrations of whole cell lysate protein were measured by Bradford assay. Twenty micrograms of total protein samples were prepared in Laemmli buffer, boiled (100 °C; 5 min) and loaded into a 10% SDS-PAGE gel. Proteins were transferred to a nitrocellulose membrane, blocked with with 5% bovine serum albumin (BSA)/Tris buffered saline with Tween 20 (TBST) (1 h at RT) and incubated overnight with anti-c-Myc antibody (Cell Signalling Technology; 1:1000; 4 °C). Blots were incubated with anti-rabbit horseradish peroxidase-conjugated secondary antibody (1:2000, Cell Signalling Technology; 45 min; RT). Blots were stripped, blocked 5% BSA/TBST (1 h; RT) and re-probed with anti-GAPDH antibody (Santa Cruz Biotechnology; 1:3000; 40 min; RT) and anti-mouse horseradish peroxidase-conjugated secondary antibody (1:2000, Cell Signalling Technology; 45 min; RT). Protein was detected with enhanced chemiluminescent substrate.

### Generation and isolation of IPE cells

Female Crl:CD1(ICR) mice (strain referred to as CD-1) were purchased from Charles River Laboratories. Adult mice between 6 and 16 weeks of age were euthanized by exposure to CO_2_ prior to tissue collection. Cervical dislocation was performed after CO_2_ asphyxiation to ensure euthanasia of mice. In each experiment primary bone marrow cells were isolated from femurs and tibias of 6–8 sacrificed mice by flushing with Hank’s balanced salt solution (HBSS) (Gibco, ref.: 14025092) supplemented with 2% fetal bovine serum (FBS) (Gibco, ref.: 12483–020), using a 23-gauge needle (BD Biosciences, ref.: 305145). The cells were pooled, centrifuged and re-suspended in 2% FBS HBSS. Existing RBCs were lysed with ammonium chloride solution (Stem Cell Technologies, ref.: 07800) and vortexing for 5 s, then incubating on ice for 5 min. The cells were then lineage-depleted (lin^−^) using a hematopoietic isolation kit (Stem Cell Technologies, ref.: 19756A) at a cell density of 2 × 10^8^ cells/ml. Purified cells were then sorted using fluorescence activated cell sorting (FACS) with conjugated antibodies and a FACSAria cell sorter (BD Biosciences) to obtain a BFU-E enriched population with surface marker profile Mac-1^−^Gr-1^−^Ter119^−^c-Kit^+^CD71^(low/−)^ [9].

BFU-E enriched cells were re-suspended in IPE culture media at 2.5 × 10^5^ cells/ml and plated in 200 μl volumes at 37 °C and 5% CO_2_ in a 96-well tissue culture plastic (TCP) plate (Sarstedt, ref.: 83.1835) to recover. One day after plating cells were transduced with the TRE3G-c-Myc lentivirus vector. One day after transduction, cells were diluted with fresh media in a 1:2 split passage, and dox (2000 ng/ml) and puromycin (1000 ng/ml) were added. Cell cultures were then passaged at a split ratio of 1:4 every 2 days, being careful not to disrupt cellular aggregates. Twenty-five days after harvest, cells were sorted by FACS using the surface marker expression profile Mac-1^−^Gr-1^−^c-Kit^(high/+)^CD71^+^. Purified cells were continuously cultured in IPE media following the same split ratio of 1:4 every 2 days in 200 μl volumes of 96 well plates making fresh IPE media with dox and puromycin for each passage. A flowchart of the IPE cell establishment and differentiation is available in the supplementary materials (Additional file 1: Figure S11).

### IPE cell differentiation assay

IPE cells were washed three times in 2% FBS in Iscove’s Modified Dulbecco’s Medium (IMDM) by centrifugation, then either plated in a 96 well TCP plate at 5 × 10^5^ cells/ml in 200 μl of IPE media or in a 6 well plate on a confluent layer of mouse stromal cells (MS-5) [41] at 1.25 × 10^5^ cells/ml in 800 μl of IPE media. The cells were left under static conditions for 2 or 4 days with no media changes before analysis. Under the two-stage differentiation protocol, the cells were transferred after 2 days of culture on TCP with low levels of dox (125 ng/ml) to a confluent layer of mouse stromal cells with 800 μl of IPE media and no dox after triple washing in 2% FBS IMDM.

### IPE media

Hematopoietic cells and IPE cells were cultured in IPE media which consists of IMDM (Gibco, ref.: 12440053) supplemented with 20% FBS (Gibco, ref.: 12483–020), 10 U/ml mouse EPO (R&D Systems, ref.: 959-ME-010), 100 ng/ml mouse SCF (R&D Systems, ref.: 455-MC-010), 500 μg/ml human holo-transferrin, 40 ng/ml human IGF-1 (R&D Systems, ref.: 291-G1–200), 10 μg/ml human insulin (Sigma, ref.: I9278), and 2x Pen/Strep (Gibco, ref.: 15070063). If necessary media was supplemented with 2 μg/ml dox (Biobasic, ref.: DB00889) to induce c-Myc expression, and/or puromycin (1000 ng/ml) (Sigma, ref.: P8833) for selection of cells containing the transfer vector.

### Lentiviral transduction of primary hematopoietic cells

Concentrated lentivirus, media supplements, and polybrene (Sigma, ref.: 107689-10G) were added to existing cell cultures at a ratio of 1:1 to achieve a final multiplicity of infection of 5, polybrene concentration of 8 μg/ml, and full IPE media supplement concentrations. The plates were centrifuged in a 32 °C pre-heated centrifuge at 1200×g for 2 h, then incubated at 37 °C and 5% CO_2_.

### Hematopoietic colony-forming cell (CFC) assays

Colony-forming cell assays [37] were performed to enumerate hematopoietic progenitor cells using Methocult M3434 (Stem Cell Technologies, ref.: 03434). For transduced cells, dox (2 μg/ml) was added and mixed when plating. In this assay, cells were mixed with M3434 solution by pipetting slowly using a blunt end needle and a syringe. The cells were then plated in a 6-well nontissue culture treated plate (Costar, ref.: 3736). Colonies were scored according to company protocol according to images provided and colony descriptions.

### Cytokine dependence assay

IPE cells were plated at 2.5 × 10^5^ cells/ml in 200 μl IPE base media (without EPO and SCF) and supplemented with various combinations of EPO and SCF in the presence of 2 μg/ml dox. Experiments were done in triplicate and cells were passaged 1:4 every 2 days. Cells were counted with a hemocytometer and trypan blue vital stain (Gibco, ref.: 15250061).

### Flow cytometry

Conjugated anti-mouse antibodies used in this study were PE-CD71 (BD, ref.: 553267), APC-c-Kit (BD, ref.: 553356), PE-Cy/7-Ter119 (BD, ref.: 557853), FITC-CD71 (BD, ref.: 553266), FITC-CD11b (Mac-1) (BioLegend, ref.: 101205), and APC-Cy/7-Ly-6G/Ly-6c (Gr-1) (Biolegend, ref.: 108423). The following nuclear dyes were used: 7-aminoactinomycin D (7-AAD) (Invitrogen, ref.: A1310), and DRAQ5 (Cell Signalling Technology, ref.: 4084S). For analytical staining, approximately 100,000 cells or less were washed with 2% FBS HBSS and incubated in a 20 μl staining volume at 4 °C between 15 min to 20 min containing relevant antibodies, each at a dilution of 1:100 in 2% FBS in HBSS. To determine viability, 7-AAD was included at a dilution of 1:500, and nuclei were stained with DRAQ5 at a dilution of 1:5000 (1 μM). After staining, cells were washed, re-suspended in 200 μl of 2% FBS HBSS, and run on a FACSCanto flow cytometer (BD Biosciences). When counting beads were used, an equal volume containing approximately 1500 blank beads (Spherotech, ref.: ACBP-20-10) was added to each sample. Fluorescence spectral overlap compensation was done using single stain compensation controls and algorithms within FlowJo (Tree Star, San Carlos, CA). A general gating strategy (Additional file 1: Figure S10) included selection of cells and beads based on their forward scatter (FSC) and side scatter (SSC), excluding highly granular cells and small debris, followed by a live/dead exclusion applied using 7-AAD.

## Additional files

Additional file 1: **Figure S1.** Detailed DNA plasmid map of lentivirus transfer vector TRE3G-cMyc. **Figure S2.** Doubling time of c-Myc knock-out HO15.19 cells modified with the TRE3G-cMyc transfer vector compared with wild-typ type cells. **Figure S3.** Fluorescence activating cell sorting gates used to isolate erythroid enriched populations from fresh lineage depleted bone marrow. **Figure S4.** Colony forming cell (CFC) results for isolated Lin^−^c-Kit^+^CD71^(low/−)^ mouse bone marrow cells. **Figure S5.** Comparing normal BFU-E and ‘BFU-E like’ colonies formed from normal and genetically modified hematopoietic cells. **Figure S6.** Cell surface protein profile of IPE cells at isolation and after culture. **Figure S7.** Cell surface protein profile of Lin^−^c-Kit^+^CD71^(low/−)^ cells modified with the TRE3G-cMyc and cultured for three weeks. Supplementary Methods. **Figure S8.** Tetramethylbenzidine (TMB) staining of IPE cells after 48 h of culture with 0 ng/ml dox. **Figure S9.** Relative size of cells comparing IPE cells, RBCs derived from IPE cells (IPE - > RBC), and fresh RBCs. **Figure S10.** Example of gating strategy based on FSC and SSC and then on 7-AAD. **Figure S11.** Flowchart for IPE cell establishment, isolation, and optimised differentiation protocol. (DOCX 3550 kb)

## Abbreviations

7-AAD: 7-aminoactinomycin D
BFU-E: Burst-forming unit-erythroid
BSA: Bovine serum albumin
CFC: Colony-forming cell
CFU-E: Colony forming unit-erythroid
DNA: Deoxyribonucleic acid
dox: doxycycline
EPO: Erythropoietin
ES: Embryonic stem
FACS: Fluorescence activated cell sorting
FBS: Fetal bovine serum
FSC: Forward scatter
GFP: Green fluorescent protein
HAT: Histone acetyl transferase
HBSS: Hanks balanced salt solution
HPV: Human papillomavirus
hUbC: Human ubiquinase C
IGF-1: Insulin like growth factor 1
IMDM: Iscove’s modified Dulbecco’s medium
IPE: Inducibly proliferating erythroid
Lin-: Lineage negative
Pen: Penicillin
Puro: Puromycin
RBC: Red blood cell
RNA: Ribonucleic acid
RT: Room temperature
rtTA: reverse tetracycline transactivator
SCF: Stem cell factor
SSC: Side scatter
strep: streptomycin
TBST: Tris-buffered saline with Tween 20
TCP: Tissue culture plate
TGF-β: Transforming growth factor beta
TRE: Tetracycline responsive element
TRE3G: Third generation TRE
U: Unit
