# Supplementary material for: Establishment of an erythroid progenitor cell line capable of enucleation achieved with an inducible c-Myc vector
Source: BMC Biotechnol. 2019 Apr 15;19:21. doi: 10.1186/s12896-019-0515-9 (PMC6466758; doi:10.1186/s12896-019-0515-9)
Supplement: Supplementary file 1 — Figure S1. Detailed DNA plasmid map of lentivirus transfer vector TRE3G-cMyc. Figure S2. Doubling time of c-Myc knock-out HO15.19 cells modified with the TRE3G-cMyc transfer vector compared with wild-typ type cells. Figure S3. Fluorescence activating cell sorting gates used to isolate erythroid enriched populations from fresh lineage depleted bone marrow. Figure S4. Colony forming cell (CFC) results for isolated Lin−c-Kit+CD71(low/−) mouse bone marrow cells. Figure S5. Comparing normal BFU-E and ‘BFU-E like’ colonies formed from normal and genetically modified hematopoietic cells. Figure S6. Cell surface protein profile of IPE cells at isolation and after culture. Figure S7. Cell surface protein profile of Lin−c-Kit+CD71(low/−) cells modified with the TRE3G-cMyc and cultured for three weeks. Supplementary Methods. Figure S8. Tetramethylbenzidine (TMB) staining of IPE cells after 48 h of culture with 0 ng/ml dox. Figure S9. Relative size of cells comparing IPE cells, RBCs derived from IPE cells (IPE - > RBC), and fresh RBCs. Figure S10. Example of gating strategy based on FSC and SSC and then on 7-AAD. Figure S11. Flowchart for IPE cell establishment, isolation, and optimised differentiation protocol. (DOCX 3550 kb) [file 12896_2019_515_MOESM1_ESM.docx]

**Supplementary Materials (Mayers et al.)**

Establishment of an erythroid progenitor cell line capable of enucleation achieved with an inducible c-Myc vector

**Supplementary Methods**

Molecular Cloning Primers

Primer Set 1:

Primer 1: GTACTAGCGGCCGCCGTGTCGGCTCCAGATCT

Primer 2: GACCTGGATATCTTACCCGGGGAGCATGTC

Primer Set 2:

Primer 1: CATATAGAGCTCACCATGGATTACAAGGACGATGACGACAAAATGCCCCTCAACGTGAA

Primer 2: CAGCCTGCGGCCGCttatgcaccagagtttcgaagc

Flag Epitope Nucleotide Sequence: GATTACAAGGACGATGACGACAAA

**Standard Cell Line Maintenance**

All adherent cell lines were generally cultured in 10% (v/v) qualified fetal bovine serum (FBS) of Canadian origin (Gibco, ref:12483-020) in Dulbecco’s Modified Eagle Medium (DMEM) (Sigma ref: D5796-500ML) without antibiotics unless otherwise specified. Cultures were done in tissue culture treated plastic vessels including 96-well plates (Sarstedt, ref: 83.1835), T-25 cell culture T-flasks (Sarstedt, ref: 83.1810), and T-75 cell cultures T-flasks (Sarstedt, ref 83.3911). Passaging included washing with Hanks Balanced Salt Solution (HBSS) (Gibco, ref: 14025092), then application of a thin layer (0.5 ml for a T25) of 0.25% Trypsin with 0.38 g/l Ethylenediaminetetraacetic acid (EDTA):4Na (Gibco, ref: 25200072) and incubated at 37 °C for a few minutes until cells detached. Cells were re-suspended in growth media (without washing) and seeded at an appropriate passage dilution, usually between 1:3 and 1:10.

**Lentivirus production**

HEK293T cells (ATCC # CRL-3216) were seeded onto T75s in base media containing 10% FBS in DMEM supplemented with 2x penicillin/streptomycin (Penn/Step) (Gibco, ref: 15070063) and 0.1% Lipogro (Rocky Mountain Biologicals, ref: LPG-BPG). After 24 hours (cells 60-80% confluent), media was replaced with base media plus 1% glutamax (Gibco, 35050-061), and 1% sodium pyruvate (Gibco, ref: 11360-070). Lipofectamine 3000 (Invitrogen, ref: L3000008) was used to transfect 20 µg of DNA into each T75-flask comprising 9.5 µg transfer vector plasmid, 7.2 µg psPAX2 packaging plasmid (Addgene: 12260) and 3.4 µg VSV-G envelope plasmid (Addgene: 12259). 24 hours after transfection, media was replaced with 10 ml of base media plus 1% glutamax, 1% sodium pyruvate, and 4 mM caffeine (Sigma, ref: C0750-5G) to increase viral titer [1]. Media was harvested and replaced at 48 and 72 hours post-transfection and stored at 4°C. Harvested media was centrifuged at 2000 × g for 4 min and passed through a 0.45 µm filter (Millipore, ref:5010-SLHA033SS), then concentrated by ultracentrifugation in a Beckman SW-28 rotor with a 2 ml 20% sucrose (Sigma, 84097-250G) cushion at 22,000 rpm (~100,000 × g) for 2 hours. Pellets were resuspended in 10% FBS DMEM and stored at -80°C.

**Lentivirus Titer Measurements**

Titer measurements were done using virus infection colony-forming assays [2] as the vectors contained a puromycin resistance gene. The adherent fibroblast cell line TGR-1 [3] was plated at 10,000 cells per well in a 96-well tissue culture treated plate. The following day, media was replaced with growth media containing various volumes of lentivirus stock and a final concentration of 8 µg/ml polybrene (Sigma, ref: 107689-10G). Cells were incubated for 24 hours to allow viral integration and expression of the puromycin resistance gene. Media was then replaced with fresh media containing puromycin at 2 µg/ml to eliminate the cells that were not transduced with the vector containing the resistance gene. Three to five days later, colonies were scored using a phase contrast microscope, where the ability of puromycin to eliminate cells that did not contain the gene vector was verified using a “no virus” control. Titers were reported as colonies per well divided by volume of viral stock used per well [2].

$$Titer=\frac{Colonies Per Well}{Volume of Viral Stock Used Per Well}$$

**Giemsa stain**

Cells were loaded into a Shandon double Cytofunnel (Fisher Scientific, ref: 2861038) with a treated glass cytoslide (Fisher Scientific, ref: 2861039), and centrifuged at 1000 rpm on a Shandon Cytospin 2 centrifuge for 10 minutes. Slides were then immediately put into 100% methanol at -20 °C for 5 min, air dried for 15 minutes at room temperature, and stained in 1:20 Giemsa stain (Sigma, ref: GS500-500ML) diluted in deionised water for 60 min at room temperature. The slide was then rinsed in deionised water and air dried before visualisation on a phase contrast microscope.

**Hemoglobin Detection with 3,3’,5,5’-Tetramethylbenzidine (TMB)**

In the presence of H_2_O_2_, hemoglobin can act as a peroxidise enzyme [4]. This peroxidase activity can be detected with colorimetric substrates such as benzidine and TMB. [5,6] Liquid substrate system (Sigma, ref: T9455-100ML) designed for membranes forms an insoluble blue product when peroxidase activity is present [7]. This reagent was added to cell cultures at an equal volume ratio of 1:1 and images after 20 minutes of reaction to detect the presence of hemoglobin. This reagent was used instead of benzidine since it is a safer alternative with equivalent specificity to the horseradish peroxide (HRP) enzymatic activity.

**Derivation of Doubling Time Calculation**

Fluorescence intensities at the initial and final time points were used to calculate a doubling time by applying simple first order ordinary differential equation exponential growth model. The development is as follows:

$$\frac{dC_{cells}}{dt}=k\cdot C_{cells}$$

Where $C_{cells}$ is the concentration of cells, $k$ is a growth constant, and $t$ is time.

Which can be solved:

$$\int\frac{dc_{cells}}{c_{cells}}=\int k\cdot dt$$

$$\ln\left( C_{cells} \right)= k\cdot t+ C_{1}$$

Applying boundary conditions where at $t=0,$ $C_{cells}$ = $C_{cells,1}$ and ${t=t}_{2},$ $C_{cells}$ = $C_{cells,2}$

$$\ln\left( C_{cells,1} \right)= k\cdot0+ C_{1}$$

$$C_{1}=\ln\left( C_{cells,1} \right)$$

Making the solution:

$$\ln\left( C_{cells,2} \right)-\ln\left( C_{cells,1} \right)= k\cdot t_{2}$$

$$\ln\left( \frac{C_{cells,2}}{C_{cells,1}} \right)= k\cdot t_{2 \boldsymbol{(}\boldsymbol{Equation} \boldsymbol{1}\boldsymbol{)}}$$

A proliferation constant, k, can be defined in terms of doubling time ($t_{doubling}$) from Equation 1

$$\ln\left( \frac{2}{1} \right)= k\cdot t_{doubling}$$

$$k=\frac{\ln(2)}{t_{doubling}}$$

Substituting k into equation 1

$$\ln\left( \frac{C_{cells,2}}{C_{cells,1}} \right)= \frac{\ln(2)}{t_{doubling}}\cdot t_{2}$$

Isolating for $t_{doubling}$

$$t_{doubling}= \frac{\ln(2)}{\ln\left( \frac{C_{cells,2}}{C_{cells,1}} \right)}\cdot t_{2}$$

And as cell density is linearly proportional to fluorescence intensity (I) [8] the following substitution can be made:

$$\frac{C_{cells,2}}{C_{cells,1}}=\frac{I_{2}}{I_{1}}$$

And then $t_{doubling}$ can be calculated using initial and final fluorescence intensities:

$$t_{doubling}= \frac{\ln(2)}{\ln\left( \frac{I_{2}}{I_{1}} \right)}\cdot t_{2}$$

Using this final equation, doubling times were calculated from initial and final fluorescence intensities for c-Myc knock-out cells, wild type cells, and modified knock-out cells containing the transfer vectors under various dox concentrations.

**Supplementary Figures**


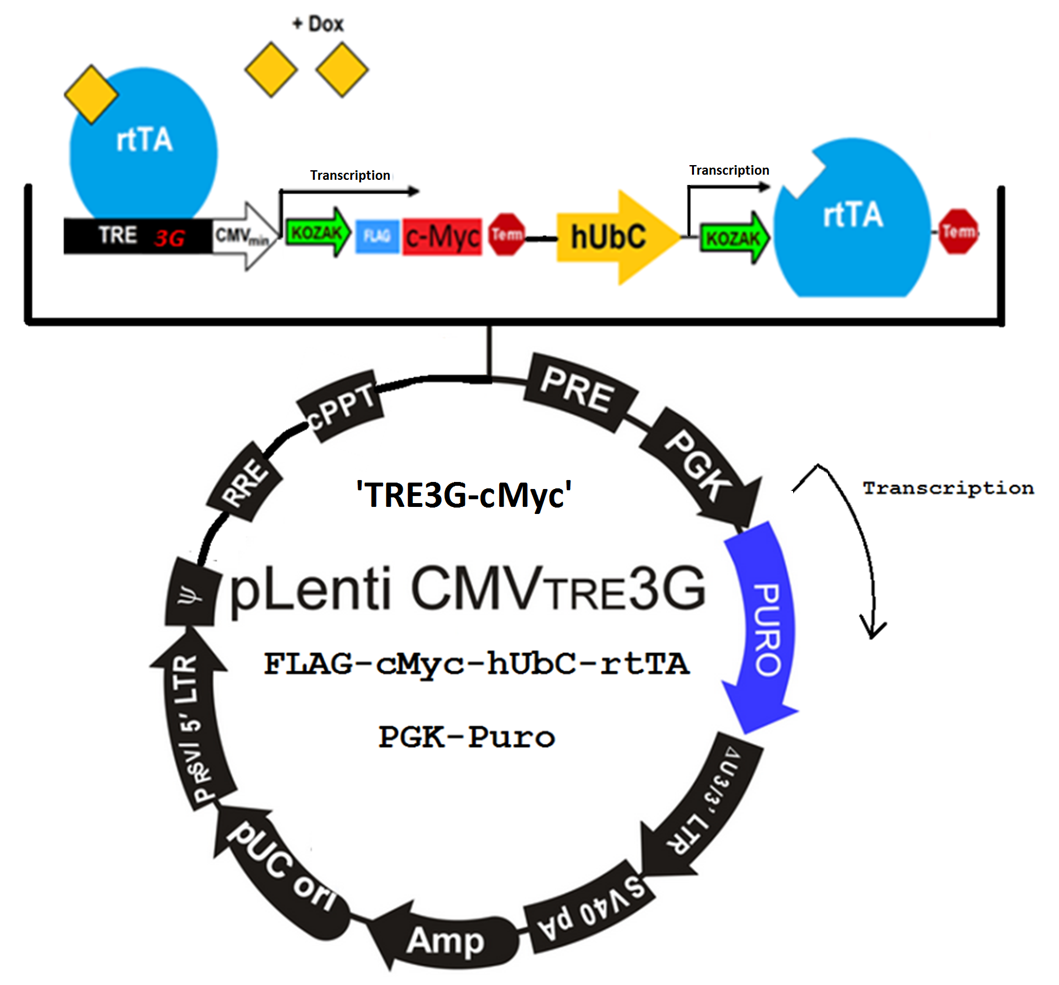


**Figure S1: Detailed DNA plasmid map of lentivirus transfer vector TRE3G-cMyc**. This third generation lentivirus transfer vector contains the third generation tetracycline responsive element (TRE3G) controlling the expression of FLAG tagged c-Myc (mouse) transcription factor, constitutive expression of the rtTA, and a puro resistance gene. Depicted is the rtTA transcription factor activated by soluble doxycycline and binding to the TRE3G promoter. The transcription initiation sites are outlined along with kozak sequences which ensure transcriptional initiation. Translational stop codons, depicted as red stop signs with the word 'term', were included at the end of the coding DNA sequence for c-Myc and rtTA. The simian virus 40 polyadenylation signal (SV40 pA), which is a common polyadenylation site for lentivirus expression vectors is included in the ΔU3/3'LTR region.


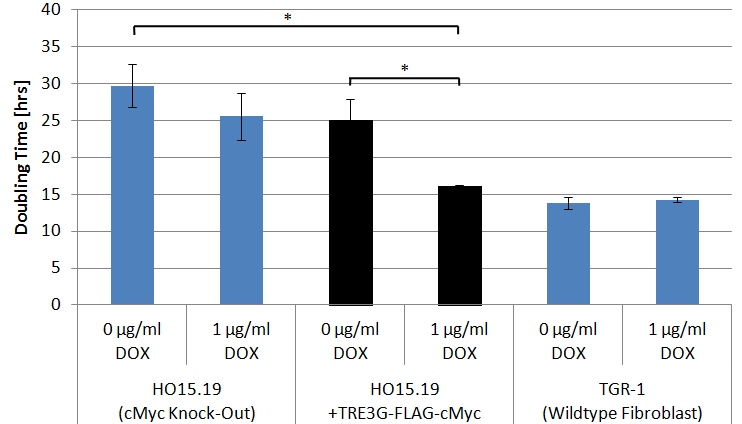


**Figure S2: Doubling time of c-Myc knock-out HO15.19 cells modified with the TRE3G-cMyc transfer vector compared with wild-typ type cells**. Assays were done by plating cells under various doxycyline conditions and measuring cell amount using AlamarBlue at 4 days. Doubling times calculated using the AlamarBlue assay assume all cells have the same reducing ability independent of genome, and cell density. Shown are the averages of 3 experiments with standard deviations indicated. Statistical tests were done by ANOVA with post hoc t-tests with Bonferroni corrections (m=6). * designates Bonferroni corrected p<0.05


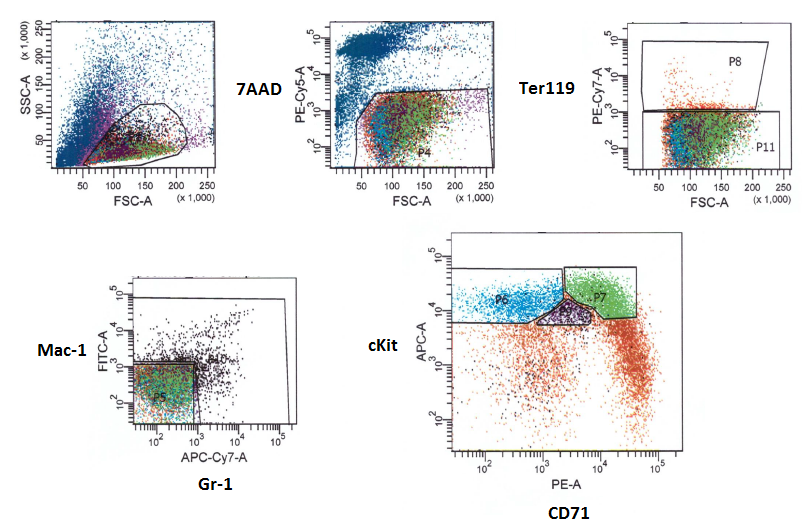


**Figure S3: Fluorescence activating cell sorting gates used to isolate erythroid enriched populations from fresh lineage depleted bone marrow.** Dead cells and debris were gated out with FSC and SSC, and a 7AAD dead cell exclusion dye. The BFU-e enriched population used in this study were Ter119^-^Mac-1^-^Gr-1^-^c-Kit^+^CD71^(low/-)^ indicated as P6.


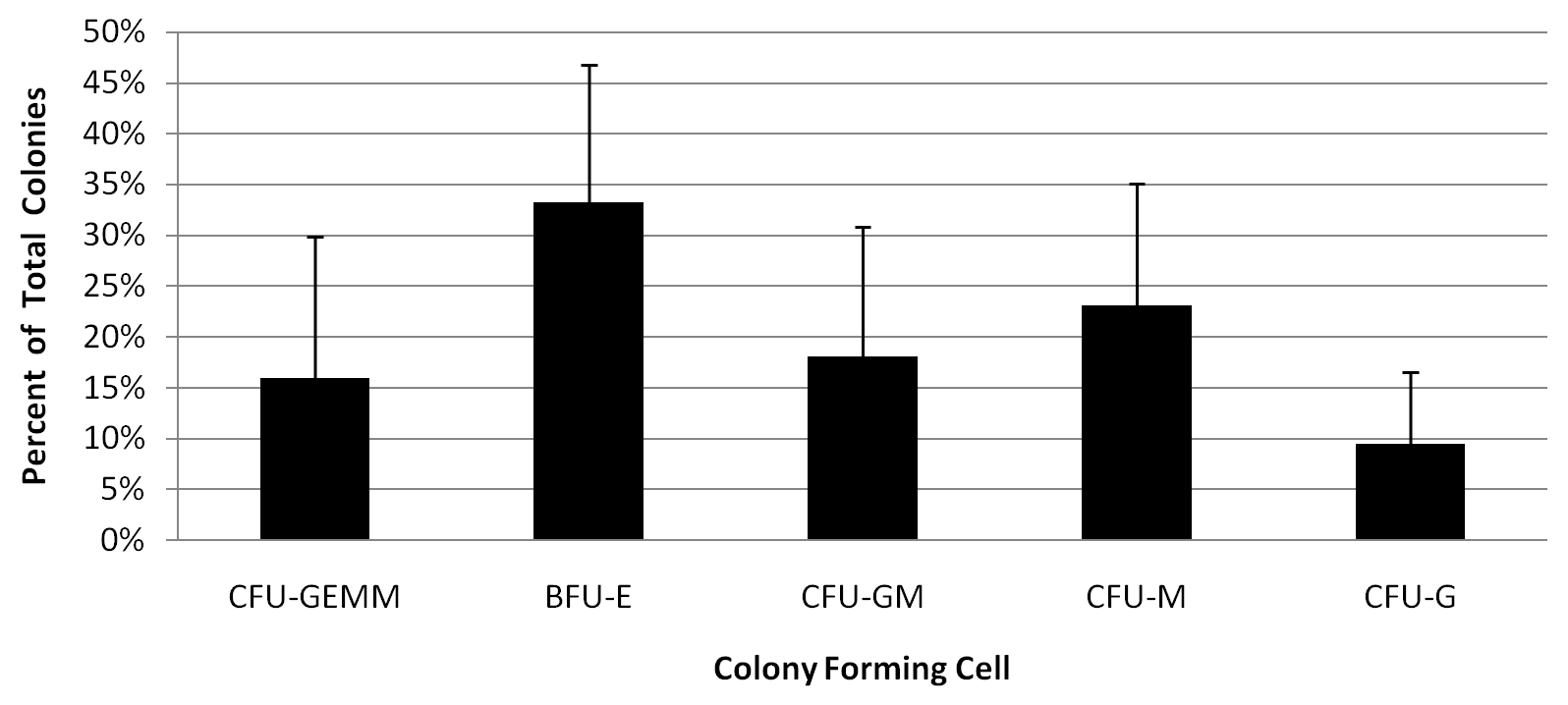


**Figure S4: Colony forming cell (CFC) results for isolated Lin^-^c-Kit^+^CD71^(low/-)^ mouse bone marrow cells**. Shown are the averages of three wells seeded with 100 cells each and cultured in M3434 immediately after harvest and FACS isolation. The colonies were counted between 1-2 weeks of culture in the methylcellulose assay. The plating efficiency of cells that formed colonies was 25 ± 3%. Shown are the averages of three independent experiments plating cells after lineage depletion and FACS.


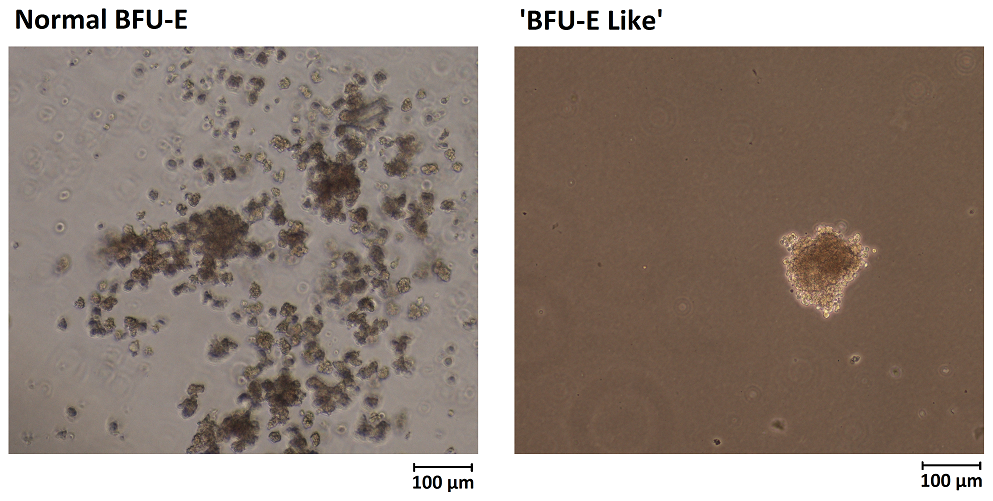


**Figure S5: Comparing normal BFU-E and 'BFU-E like' colonies formed from normal and genetically modified hematopoietic cells**. 'BFU-E Like' colonies were generated from cells modified with the FLAG-c-Myc transfer vector and ectopically expressing c-Myc by addition of 2 µg/ml dox. These large clusters of small closely packed cells are atypical for mouse hematopoeitic cells, as CFU-Es are generally smaller clusters and BFU-Es are more dispersed. The 'BFU-E like' colony resembles human BFU-Es as described in the Stem Cell Technologies Handbook [9].


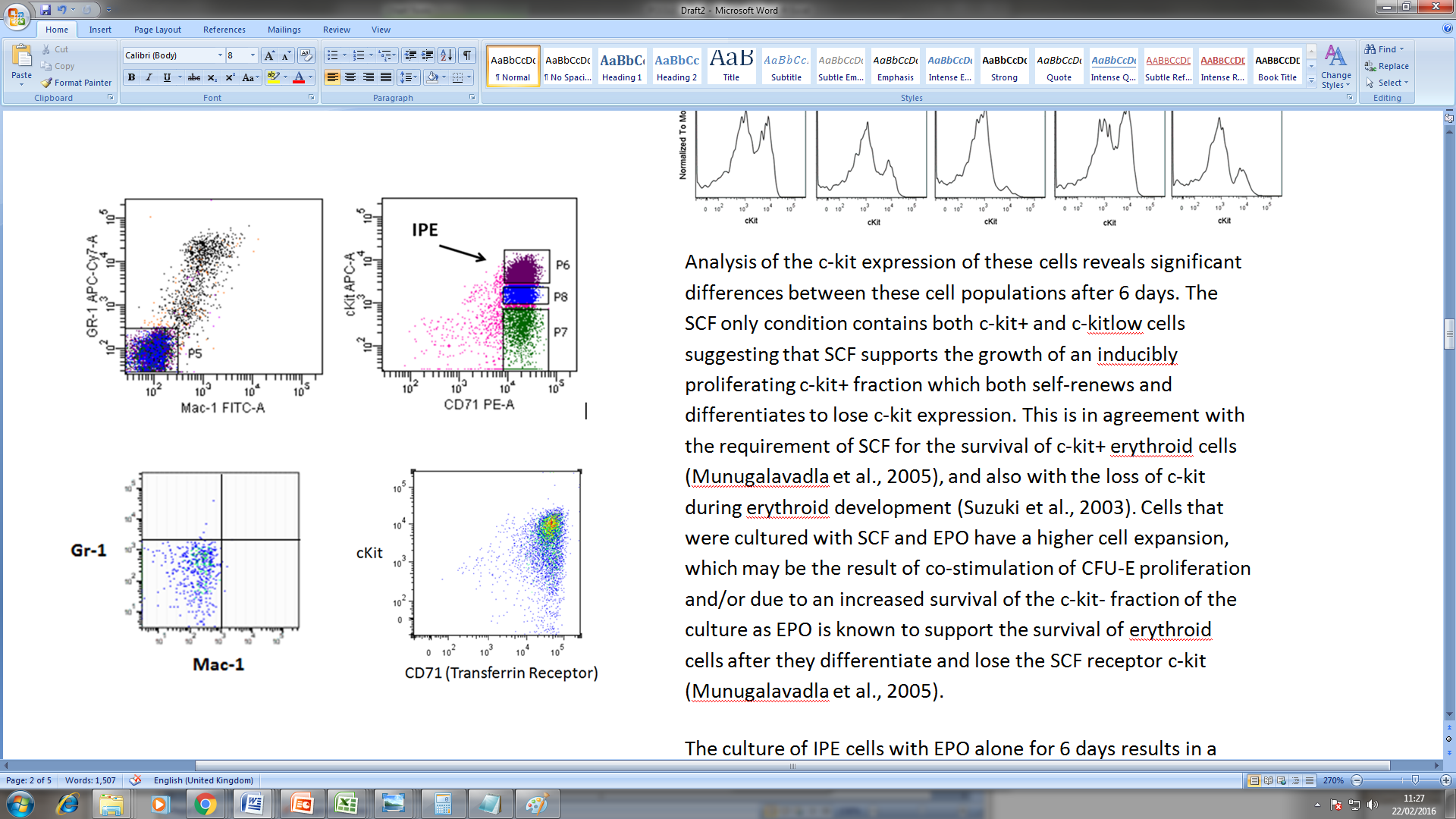


**Figure S 6: Cell surface protein profile of IPE cells at isolation and after culture.** (TOP) FACS profile for TRE3G-cMyc vector modified Lin^-^c-Kit^+^CD71^(low/-)^ cells purified with 1 µg/ml puromycin, and cultured with 2 µg/ml dox for 3.5 weeks from harvest. Gr-1^+^ and Mac-1^+^ cells were removed using gate P5, and a c-Kit^+^CD71^+^ population (P6) was isolated and designated IPE cells. (BOTTOM) IPE cells 23 days after sorting (48 days after harvest) and being cultured in IPE base media being passaged 1:4 every 2 days. Cells were stained with conjugated antibodies specific to c-Kit, CD71, Mac-1, and Gr-1 and appear to have the same phenotype as those collected with FACS with the exception that significant c-Kit^-^ cells were present.


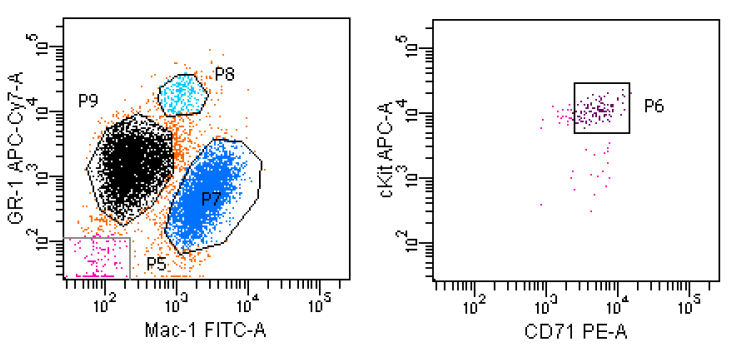


**Figure S7: Cell surface protein profile of Lin^-^c-Kit^+^CD71^(low/-)^ cells modified with the TRE3G-cMyc and cultured for three weeks.** Flow cytometry profile for TRE3G-cMyc vector modified Lin^-^c-Kit^+^CD71^(low/-)^ cells modified with the TRE3G-cMyc vector purified with 1 µg/ml puromycin, and cultured with 2 µg/ml dox for three weeks. Dead cells and debris were gated out with forward and side scatter measurements as well as 7AAD staining. A high proportion of Gr-1^+^ and Mac-1^+^ cells were observed in this experiment.


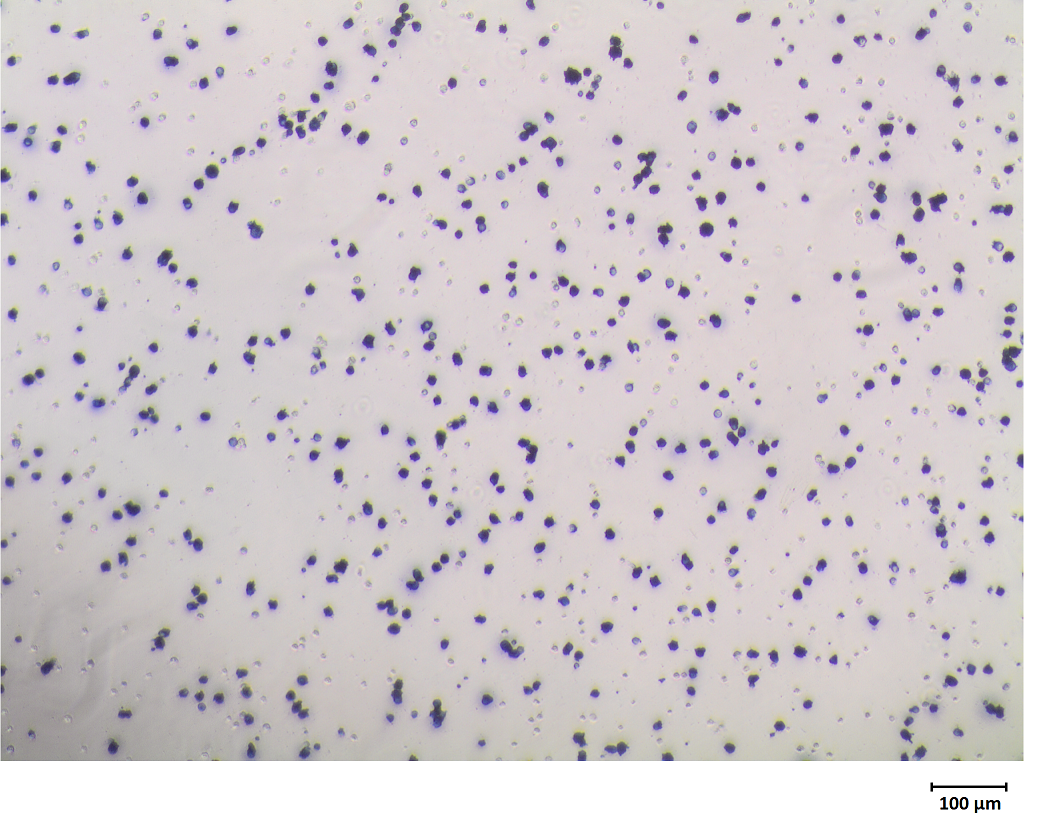


**Figure S8: Tetramethylbenzidine (TMB) staining of IPE cells after 48 hours of culture with 0ng/ml dox.** Phase contrast image (100x magnification) of IPE cells after incubation with normal IPE cell media on TCP with 0ng/ml dox for 48 hours. Cells were stained with TMB, which forms an insoluble blue product in the presence of hemoglobin through its horseradish peroxide (HRP) enzymatic activity.


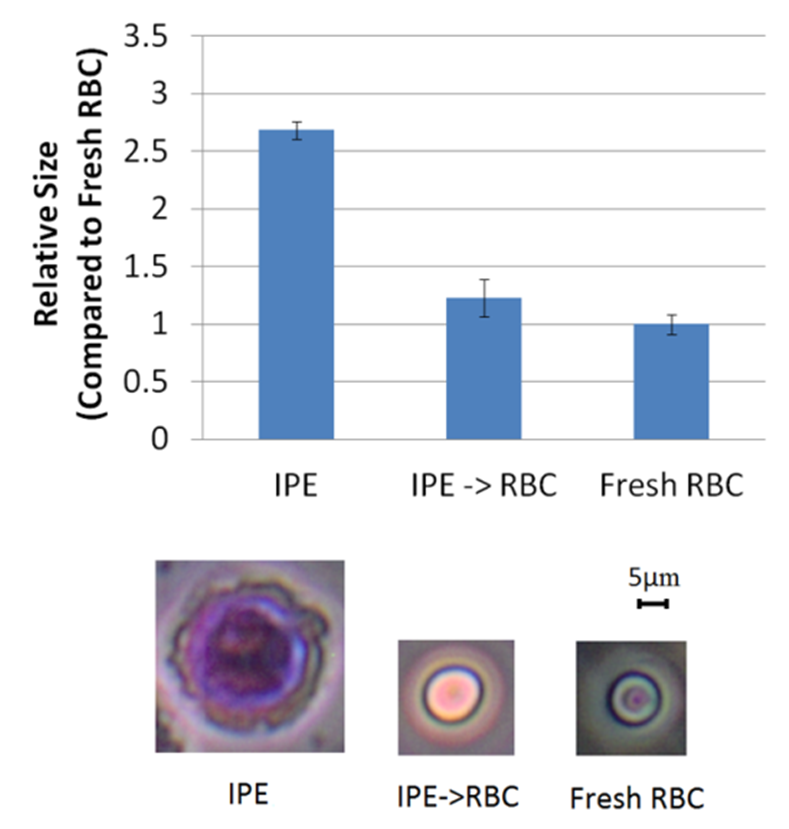


**Figure S9: Relative size of cells comparing IPE cells, RBCs derived from IPE cells (IPE -> RBC), and fresh RBCs.** Relative size of the cell diameter was the measured from phase contrast microscope images at 400x magnification. IPE cells were imaged after Giemsa histology stain, where fresh wildtype RBCs were imaged without staining.


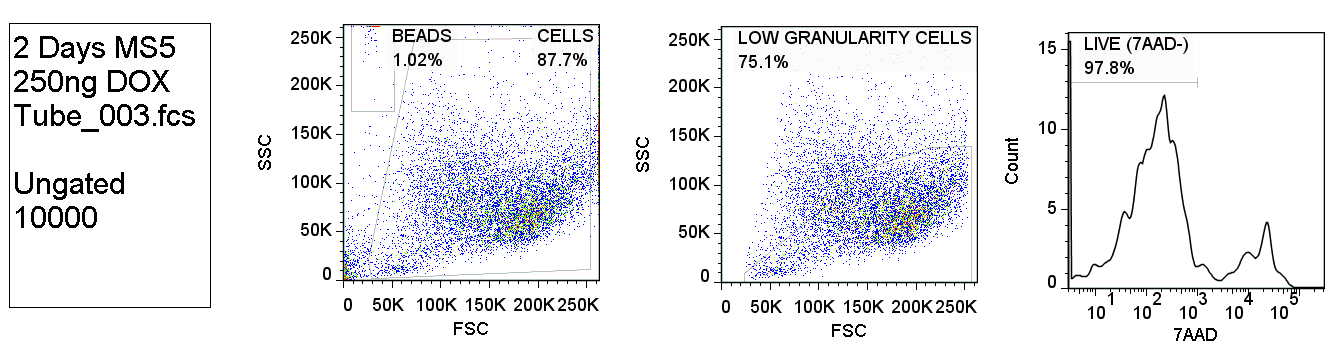


**Figure S10: Example of gating strategy based on FSC and SSC and then on 7-AAD.** In this representative sample, the first gates exclude very small debris and cell counting beads, the second gate excludes granular cells, and the third gate excludes dead cells containing the dye 7AAD. All the events in the final population are likely to be live cells which were analysed for surface antigen expression, or with a cell permeable nuclear stain.


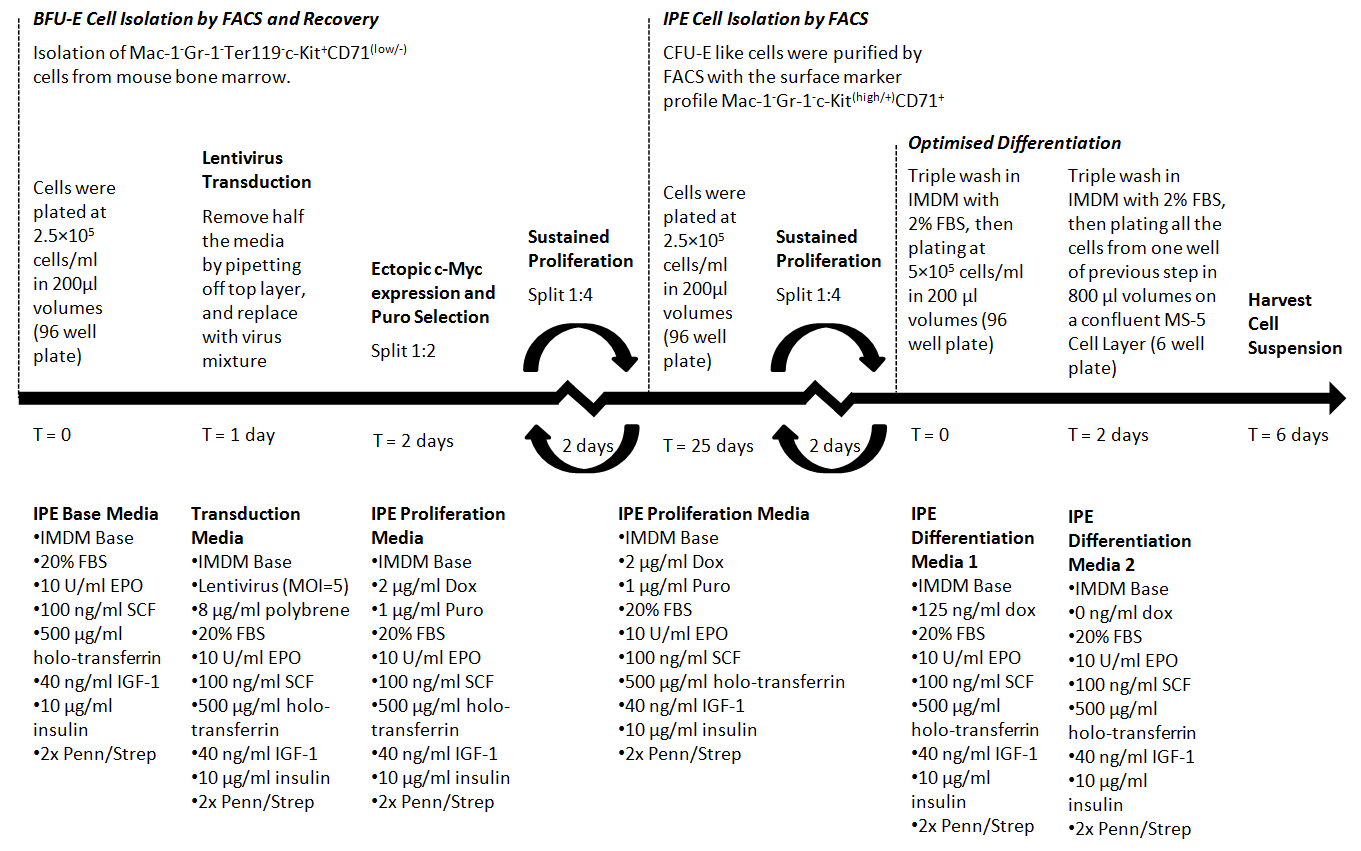
**Figure S11: Flowchart for IPE cell establishment, isolation, and optimised differentiation protocol.** Key stages are outlined through the course of the experiments, including BFU-E cell isolation, lentivirus transduction, ectopic gene expression and purocymin (Puro) selection, sustained proliferation, IPE cell isolation, and optimised differentiation. Media composition is included for each process stage as well as passage split ratios, seeding densities and cell washing steps.

**Supplementary Materials References**

1. Ellis BL, Potts PR, Porteus MH. Creating higher titer lentivirus with caffeine. Hum Gene Ther. 2011;22:93–100.

2. Burns JC, Friedmann T, Driever W, Burrascano M, Yee JK. Vesicular stomatitis virus G glycoprotein pseudotyped retroviral vectors: concentration to very high titer and efficient gene transfer into mammalian and nonmammalian cells. Proc Natl Acad Sci U S A. 1993;90:8033–7.

3. Mateyak MK, Obaya a J, Adachi S, Sedivy JM. Phenotypes of c-Myc-deficient rat fibroblasts isolated by targeted homologous recombination. Cell Growth Differ. 1997;8:1039–48.

4. Kapralov A, Vlasova II, Feng W, Maeda A, Walson K, Tyurin VA, et al. Peroxidase activity of hemoglobin·haptoglobin complexes. Covalent aggreation and oxidative stress in plasma and macrophages. J Biol Chem. 2009;284:30395–407.

5. Liem HH, Cardenas F, Tavassoli M, Poh-Fitzpatrick MB, Muller-Eberhard U. Quantitative determination of hemoglobin and cytochemical staining for peroxidase using 3,3???,5,5???-tetramethylbenzidine dihydrochloride, a safe substitute for benzidine. Anal Biochem. 1979;98:388–93.

6. Reynolds M, Lawlor E, McCann SR, Temperley I. Use of 3,3’,5,5’-tetramethylbenzidine (TMB) in the identification of erythroid colonies. J Clin Pathol. 1981;34:448–9.

7. Sigma-Aldrich. Product Information: TMB Enhanced One Component HRP Membrane Substrate (Catalog Number: T9455) [Internet]. Tech. Bull. Sigma-Aldrich Co. LLC. 2012 [cited 2018 Oct 14]. p. 1–2. Available from: https://www.sigmaaldrich.com/content/dam/sigma-aldrich/docs/Sigma/Bulletin/1/t9455bul.pdf

8. Stro T, Dimberg A, Hammarberg A, Carlson K, O A, Nilsson K, et al. Rapamycin sensitizes multiple myeloma cells to apoptosis induced by dexamethasone. Cancer. 2004;103:3138–47.

9. Technologies SC. Human Colony-Forming Unit (CFU) Assays Using MethoCult^TM^ [Internet]. Tech. Man. 2012 [cited 2018 Apr 2]. p. 25–25. Available from: https://cdn.stemcell.com/media/files/manual/MA28404-Human_Colony_Forming_Unit_Assays_Using_MethoCult.pdf?_ga=2.133015950.1006677047.1522696582-2146977796.1522600931
